# SARS-CoV-2 and the role of orofecal transmission: a systematic review

**DOI:** 10.12688/f1000research.51592.1

**Published:** 2021-03-24

**Authors:** Carl J. Heneghan, Elizabeth A. Spencer, Jon Brassey, Annette Plüddemann, Igho J. Onakpoya, David H. Evans, John M. Conly, Tom Jefferson

**Affiliations:** 1 Centre for Evidence Based Medicine, University of Oxford, Oxford, OX2 6GG, UK; 2 Trip Database Ltd, Newport, NP20 3PS, UK; 3 Department of Medical Microbiology & Immunology, Li Ka Shing Institute of Virology, University of Alberta, Edmonton, Alberta, T6G 2E1, Canada; 4 Alberta Health Service, University of Calgary, Calgary, T2N 4Z6, Canada

**Keywords:** Orofecal, transmission, COVID-19, SARS-CoV-2, systematic review

## Abstract

**Background: **Mode of transmission of SARS-CoV-2 is of key public health importance. SARS-CoV-2 has been detected in the feces of some COVID-19 patients, suggesting the possibility that the virus could, in addition to droplet and fomite transmission, be transmitted via the orofecal route.

**Methods: **This review is part of an Open Evidence Review on Transmission Dynamics of COVID-19. We conduct ongoing searches using WHO COVID-19 Database, LitCovid, medRxiv, and Google Scholar; assess study quality based on five criteria and report important findings on an ongoing basis. Where necessary, authors are contacted for further details on the content of their articles.

**Results: **We include searches up until 20 December 2020. We included 110 relevant studies: 76 primary observational studies or reports, and 35 reviews (one cohort study also included a review) examining the potential role of orofecal transmission of SARS-CoV-2. Of the observational studies, 37 were done in China. A total of 48 studies (n=9,081 patients) reported single cases, case series or cohort data on individuals with COVID-19 diagnosis or their contacts and 46 (96%) detected binary RT-PCR with 535 out of 1358 samples positive for SARs-CoV-2 (average 39.4%). The results suggest a long duration of fecal shedding, often recorded after respiratory samples tested negative, and symptoms of gastrointestinal disease were reported in several studies. Twenty-nine studies reported finding SARS-CoV-2 RNA in wastewater, river water or toilet areas. Six studies attempted viral culture from COVID-19 patients’ fecal samples: culture was successful in 3 of 6 studies, and one study demonstrated invasion of the virus into the intestinal epithelial cells.

**Conclusions: **Varied observational and mechanistic evidence suggests SARS-CoV-2 can infect and be shed from the gastrointestinal tract, including some data demonstrating viral culture in fecal samples. Future studies should test this hypothesis rigorously to allow the development of appropriate public health measures.

## Introduction

Understanding how, when and in what types of settings SARS-CoV-2 spreads between people is critical to developing effective public health and infection prevention and control measures to break the chains of transmission. Current evidence suggests SARS-CoV-2 is primarily transmitted via respiratory droplets and fomites between infected individuals and others in close contact ^1^.

SARS-CoV-2 has been shown to contaminate and survive on certain surfaces, but currently, no reports have directly demonstrated fomite to human transmission. SARS-CoV-2 has also been detected in the feces of some patients which suggests the possibility of fomite transmission and that SARS-CoV-2 could transmit via the orofecal route. It is well recognized that coronaviruses are major pathogens in many mammalian species and predominantly target epithelial lining cells in the respiratory and gastrointestinal (GI) tracts. Many animal coronaviruses are transmitted by the fecal-oral route and there are many reports of intestinal disease associated with SARS-CoV-1 and other human coronaviruses. Main causes include lack of adequate sanitation and poor hygienic practices. Fecal contamination of food is another form of orofecal transmission. Therefore, we aimed to systematically review the evidence on orofecal SARS-CoV-2 transmission. Terminology for this article can be found in Box 1.

**Box 1. Terminology**

**Orofecal:** describes a route of transmission where the virus in fecal particles can pass from one person to the mouth of another.

**Viral load:**A measure of the number of viral particles present in an individual.

**Cycle threshold:** The number of cycles required for the fluorescent signal to cross the threshold. Ct levels are inversely proportional to the amount of target nucleic acid in the sample.

## Methods

We are undertaking an open evidence review investigating factors and circumstances that impact on the transmission of SARS-CoV-2, based on our protocol (see *Extended data*: Appendix 1 ^2^). For the original protocol, see https://www.cebm.net/evidence-synthesis/transmission-dynamics-of-covid-19/. In brief, this review aims to identify and evaluate relevant articles (peer-reviewed or awaiting peer review) that examine the mode of viral transmission and ecological variables influencing the mode of transmission. We conduct an ongoing search using WHO COVID-19 Database, LitCovid, medRxiv and Google Scholar for keywords and associated synonyms. Results are reviewed for relevance and for articles that looked particularly relevant forward citation matching was undertaken and relevant results were identified. Studies with modelling are only included if they report transmission outcome data and not predicted outcomes (see further details of the search strategy in the *Extended data:* Appendix 2 ^2^). Searches are updated every two weeks.

We extracted data on the type of study, setting, sample source and methods, fecal PCR positive samples for SARS-CoV-2 RNA including cycle threshold (including methods), symptom chronology in relation to PCR testing and/or taking samples and viral culture. We tabulated the data and summarised data narratively by mode of sample. We assessed quality using a modified QUADAS-2 risk of bias tool ^3^. We simplified the tool as the included studies were not designed as primary diagnostic accuracy studies and assessed study quality based on five criteria. Where necessary we write to authors of included studies for further details or clarification on the content of their articles. Meta-analyses were not performed, due to the variability of available data. The protocol was last updated on 1 December 2020 ( Version 3: 1 December 2020).

## Results

**Figure 1.**
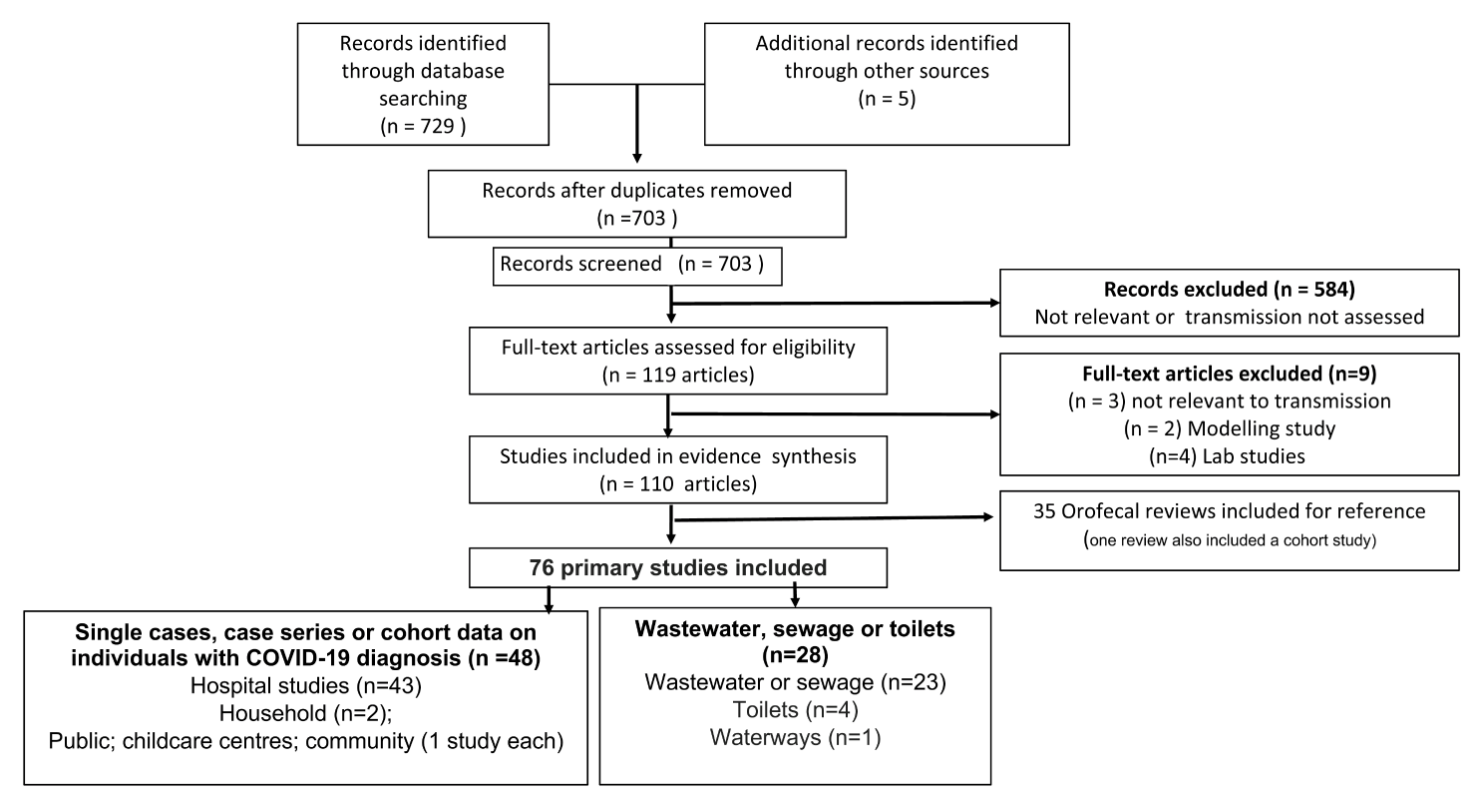
Study flow diagram.

This update includes searches up until 20 December 2020 (see Figure 1). We identified 110 relevant studies (see *Extended data:* Appendix 3 for references of included studies ^2^): 76 primary studies or reports, and 35 reviews, one of which also reported primary study results from a cohort study [Cheung K 2020].

### Reviews

The included reviews summarised a range of observational studies including studies of detection of SARS-CoV-2 RNA in fecal samples of individuals testing positive for SARS-CoV-2 in respiratory samples, frequency of GI symptoms among those with COVID-19, and observations of SARS-CoV-2 RNA in toilets and wastewaters. The reviews included overlapping studies and must therefore not be considered as entirely additional information. Five followed systematic review methodology and reporting [Edwards 2020, Karia 2020, Pamplona 2020, Parasa 2020, Santos 2020]. The quality of the other reviews was low to moderate, with none assessing included study quality, and with reporting of methods often missing or very limited.

None of these reviews focussed on the viability (and hence transmission potential) of SARS-CoV-2 identified in fecal or wastewater samples. A review on the potential for foodborne transmission of SARS-CoV-2 found no published studies of SARS-CoV-2 survival in or on food products. The totality of the reviews’ evidence shows that the SARS-CoV-2 RNA is commonly present in stool samples of COVID-19 patients but it is unknown if this represents primary invasion of enterocytes or simply saliva and sputum that has been swallowed and is transiting it way through the GI tract. The presence of viral RNA in the feces does not allow any conclusions to be drawn about infectiousness. The contribution of orofecal transmission to viral spread in the pandemic has not been established or quantified.

### Primary studies

**Figure 2.**
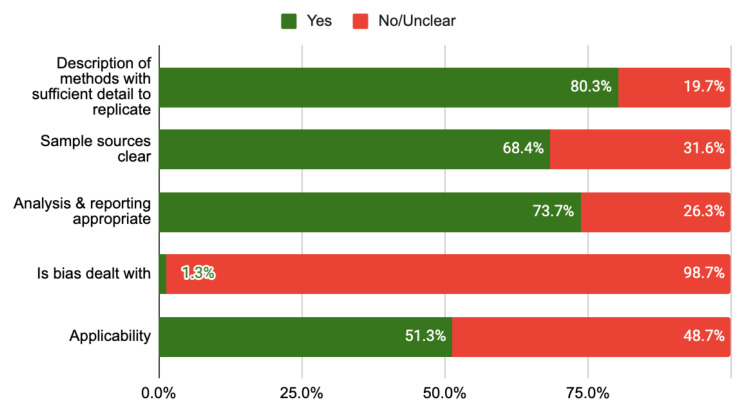
Risk of bias chart.

***Quality of included studies.*** Overall the quality of the evidence was low to moderate mainly due to a lack of standardisation of techniques, omissions in reporting and a failure to account for biases in the research process (see Table 3; Figure 2). Sample sources were clear in two-thirds of studies (65.8%). Several studies mention the possibility of bias influencing their findings but did not use strategies (design or analysis) to deal with bias, and as such have been recorded as unclear risk of bias.

***Results.*** A total of 48 (n=9,081 patients) studies reported single cases, case series or cohort data on individuals with COVID-19 diagnosis or their contacts and 46 studies (96%) detected binary RT-PCR with 535 out of 1358 samples RT-PCR positive for SARS-CoV-2 (average 39.4%). All but five studies were in hospitalized patients; 31 were done in China; the others were in East Asia, South East Asia, South Asia, USA and Europe (see Table 1).

**Table 1.** Included study characteristics: primary studies.

Study ID cohorts or case series (n=48)	Setting	Country	Population/ environment	Patient numbers	Fecal samples PCR- positive n/d for SARS-CoV-2 RNA unless otherwise stated	Live culture (positivity rate)	Genome sequencing methods	Genome Sectioning /phylogeny results where available
Chan 2020	Household	China	Family members	6	0/6	Not attempted	Yes	Two complete virus genomes (HKU-SZ-002a and HKU-SZ-005b) were sequenced & showed a novel coronavirus that is most closely related to those of a bat SARS-like coronavirus. Exploratory WGS so not confirming similarity with previous studies.
Chen C 2020	Hospital	China	121 adults, 22 children	133	22/133	Not attempted	no	
Chen W 2020	Hospital	China	Adult patients with pneumonia	57	11/28	Not attempted	no	
Chen Y 2020	Hospital	China	Hospitalised adult patients	42	28/42	Not attempted	no	
Cheung CCL 2020	Hospital	Singapore	Hospitalised adult patients	2	N/A	Not attempted	no	
Cheung K 2020	Public	Hong Kong	Cohort and systematic review	52	9/52	Not attempted	no	
Cho 2020	Hospital paediatric department	S Korea	Case study of hospitalised infant	1	1/1	Not attempted	no	
Chu H 2020	Hospital	China	Case study of breastfeeding woman	1	1/1	Not attempted	no	
COVID Ix Team	Hospital and community	USA	Patients in six US states	12	7/10	Not attempted	no	
Ge 2020	Hospital	Japan	Case reports of one hospitalised Covid-19 patient	1	1/1	Not attempted	no	
Han C 2020	Hospital	China	Patients diagnosed with low severity Covid-19	206	12/22	Not attempted	no	
Hayee 2020	Hospital outpatients	UK	Patients (non-Covid- 19) attending for GI endoscopy	6,208	N/A	Not attempted	no	
Hoehl S 2020	Childcare centres	Germany	Children and staff of childcare settings	1,197	1/5,907	Not attempted	no	
Holshue 2020	Hospital	USA	Case report	1	1/1	Not attempted	no	
Jeong 2020	Hospital	Korea	Specimens from 5 patients positive by qPCR	5	5/5	yes (0/3)	no	
Jiehao 2020	Children's hospital	China	Children	10	5/6	Not attempted	no	
Kim J-M 2020	Hospital	South Korea	Hospitalised adult patients	74	8/74	yes (0/13)	no	
Lescure 2020	Hospital	France	Case series of hospitalised adult patients	5	2/5	Not attempted	no	
Li 2020	Hospital	China	Case series of hospitalised adult patients	29	4/29	Not attempted	no	
Ling 2020	Hospital	China	66 recovered patients	66	11/28	Not attempted	no	
Lo 2020	Hospital	Macau, China	Hospitalised adult patients	10	10/10	Not attempted	no	
Nicastri 2020	Hospital	Italy	Community	1	1/1	Not attempted	no	
Pan 2020	Hospital	China	Hospitalised adult patients	17	9/17	Not attempted	no	
Peng 2020	Hospital	China	Hospitalised adult patients	9	2/9	Not attempted	no	
Qian 2020	Hospital	China	A case report of a patient admitted	1	0/1	No, but virions observed	no	
Senapati 2020	Hospital	India	Hospitalised adult patients	12	12/12	Not attempted	no	
Tan 2020	Hospital	Vietnam	Hospitalised adult patient	1	1/1	Not attempted	no	
Tang 2020	Community	China	Asymptomatic child	1	1/1	Not attempted	no	
Wang Q-X 2020	Hospital	China	Covid-19 patients with -ve respiratory tract but +ve fecal samples	5	5	Not attempted	no	
Wang S 2020	Hospital	China	Retrospective study of 17 hospitalised Covid- 19 patients	17	11	Not attempted	no	
Wang W, Xu Y 2020	Hospital	China	Patients in three hospitals	205	1/6	yes (2/4)	no	
Wang X, Zheng J 2020	Hospital	China	Hospital Covid-19 patients	69	20/69	Not attempted	no	
Wang X, Zhou Y 2020	Hospital	China	Case reports on readmitted adult	3	3/3	Not attempted	no	
Wolf 2020	Household	Germany	Family cluster	5	2/5	Not attempted	no	
Wölfel 2020	Hospital	Germany	Hospitalised adult patients	9	9/9	yes (0/13)	no	
Wu Y 2020	Hospital	China	Hospitalised adult patients	74	41/74	Not attempted	no	
Xiao F, Tang M 2020	Hospital	China	Hospitalised children and adults	71	39/73	Not attempted	no	
Xiao F, Sun J 2020	Hospital	China	Hospitalised adult patients	28	12/28	yes (2/3)	Yes	Obtained full-length viral genome sequence (GenBank accession no. MT123292) by using next-generation sequencing. The sequenced showed 5 nt substitutions compared with the original Wuhan strain (GenBank accession no. NC045512.2)
Xing Y 2020	Paediatric hospital	China	Children	3	3/3	Not attempted	no	
Xu Y 2020	Hospital	China	Children	10	8/10	Not attempted	no	
Yang 2020	Hospital	China	Paediatric Covid-19 patients	35	17/35	Not attempted	no	
Young 2020	Hospital	Singapore	Hospitalised adult patients	18	4/8	Not attempted	no	
Yuan 2020	Hospital	China	Children	78	37/78	Not attempted	no	
Zhang J 2020	Hospital	China	Patients with Covid-19 pneumonia.	14	14/14	Not attempted	no	
Zhang T 2020	Public hospital	China	Children post discharge	3	3/3	Not attempted	no	
Zhang W 2020	Pulmonary hospital	China	Hospitalised adult patients	15	4/15	Not attempted	no	
Zhang Y, Chen C, Zhu S 2020	Hospital	China	Adult severe pneumonia case report	1	1	yes (1/1)	Yes	full-length genome sequence on the one specimen using ABI 3130 Genetic Analyzer
Zhang Y, Chen C, Song Y 2020	Hospital	China	PCR-confirmed Covid-19 patients with clinical symptoms	258	93	[reports same live culture as above report]	Yes	full-length genome sequence on the one specimen using ABI 3130 Genetic Analyzer
Sewage				9081				
Agrawal 2020	Wastewater treatment plants	Germany	Two wastewater influent sources	N/A	Sewage	Not attempted	no	
Ahmed 2020	Sewage	Australia	Wastewater	N/A	Sewage	Not attempted	no	
Ampuero 2020	Two wastewater treatment plants	Chile	Wastewater	N/A	Sewage	Not attempted	no	
Arora 2020	Wastewater	India	14 wastewater treatment plants	N/A	Sewage	Not attempted	no	
Betancourt 2020	Wastewater	USA	University campus wastewater system	N/A	Wastewater	Not attempted	no	
Chavarria-Miro 2020	Sewage	Spain	Wastewater	N/A	Sewage	Not attempted	no	
Curtis 2020	Wastewater	USA	Community wastewater services	N/A	Wastewater	Not attempted	no	
Fongaro 2020	Sewage	Brazil	Urban sewage service	N/A	Sewage	Not attempted	no	
Fernandez-de-Mera 2020	Wastewater	Spain	Village community and wastewater	N/A	Wastewater	Not attempted	no	
Haramoto 2020	Wastewater and river water	Japan	Wastewater and river water	N/A	Wastewater only (river samples negative)	Not attempted	no	
Hata 2020	Wastewater	Japan	Wastewater	N/A	Wastewater	Not attempted	no	
Iglesias 2020	Urban raw surface water contaminated with wastewater	Argentina	Raw surface water samples in a low- income urban community	N/A	Wastewater- contaminated surface water	Not attempted	no	
Izquierdo-Lara 2020	Sewage	Belgium & The Netherlands	Sewage from a number of sewage facilities	N/A	Sewage	Not attempted	Yes	To further investigate genetic diversity of SARS-CoV-2, 55 wastewater samples from 13 different locations in the Netherlands (48 samples) and 7 different locations in Belgium (7 samples) with Ct values of <36 were selected for whole genome sequencing using Nanopore sequencing.
La Rosa 2020b	Sewage	Italy	Water environments	N/A	Sewage	Not attempted	no	
Medema 2020	Sewage	The Netherlands	Public, cities and airport	N/A	Sewage	Not attempted	no	
Neault 2020	Wastewater	Canada	Wastewater samples from two urban wastewater recovery facilities	N/A	Wastewater	Not attempted	no	
Peccia 2020	Sewage	USA	Primary sewage sludge sampled over time	N/A	Sewage	Not attempted	no	
Sharif 2020	Wastewater	Pakistan	Wastewater & drainage samples from across the country	N/A	Wastewater	Not attempted	no	
Shutler 2020	Waterborne	32 countries	Faecal contaminated water	N/A	Sewage	Not attempted	no	
Trottier 2020	Wastewater	France	Urban wastewater treatment plant	N/A	Wastewater	Not attempted	no	
Wang J 2020	Hospital	China	Surfaces and sewage samples	N/A	Sewage	yes (0/5)	no	
Wurtzer 2020	Community	France	Wastewater	N/A	Sewage	Not attempted	no	
Zhao 2020	Wastewater systems	China	Municipal and hospital wastewater systems	N/A	influent, effluent, sludge	Not attempted	no	
Toilets (n=4)								
Del Brutto 2020	Community	Guatemala	Rural low-income village community	362	N/A	Not attempted	no	
Ding Z 2020	Infectious diseases hospital	China	Surface samples	N/A	Toilet samples	Not attempted	no	
Kang 2020	Building plumbing system	China	Residential high-rise apartment block	N/A	Air samples around drainage system	Not attempted	no	
Ong 2020	Hospital	Singapore	Sampling in the physical areas around patients	3	Sewage toilet samples	Not attempted	no	
Waterways (n=1)								
Guerrero-Latorre 2020	Rivers	Guatemala	Urban streams in low sanitation setting	N/A	River samples	Not attempted	no	

***PCR testing for SARS-CoV-2 RNA in fecal samples in hospitalised COVID-19 patients.*** Of the 43 hospital studies, 41 (95%) detected binary RT-PCR, with 522 positive tests out of 1293 fecal samples (average 40.4%) from COVID-19 patients (see Table 4). These studies were mainly small case series, they included patients of a range of ages from infant to elderly and with widely varying severity of the disease, and the proportion of fecal samples varies from 1 (nine studies) to 258 [Zhang Y, Chen C], and the proportion testing positive for SARS-CoV-2 RNA varied from 14% to 100% across studies. One study that identified SARS-CoV-2 RNA in fecal samples among 39 of 73 hospitalised COVID-19 patients, also studied the gastric, duodenal and rectal epithelia of one patient using specimens collected via endoscopy [Xiao F, Tang M 2020].

Immunofluorescence data showed that ACE2 protein, proven to be a cell receptor for SARS-CoV-2, was abundantly expressed in the glandular cells of gastric, duodenal, and rectal epithelia, supporting entry of SARS-CoV-2 into the host cells. Intracellular staining of viral nucleocapsid protein in gastric, duodenal, and rectal epithelium showed that SARS-CoV-2 infects these GI glandular epithelial cells. Viral RNA was also detected in esophageal mucous tissue, but a lack of viral nucleocapsid protein staining in esophageal mucosa suggested low viral infection there. Viral nucleocapsid protein in rectal epithelial cells was detected in specimens from some additional COVID-19 patients, suggesting that infectious SARS-CoV-2 can survive the GI environment.

### GI symptoms among COVID-19 patients

Reporting of GI symptoms among COVID-19 patients is frequent but not consistent within these studies (diagnosis is typically based on fever, respiratory symptoms and the results of PCR testing in respiratory swabs, so recording GI symptoms may not be routine). However, several observational studies report the presence of GI symptoms among COVID-19, including Chan 2020, Cheung 2020 and Han C 2020. GI symptoms do not necessarily correlate in severity or time with other COVID-19 disease symptoms. 

### Timing and duration of fecal shedding

Fecal shedding of SARS-CoV-2 has been reported throughout the disease course and also continuing after respiratory samples tested negative. A five-person family with a confirmed COVID-19 case was hospitalized and observed: the parents and two children aged two and five years became infected but the youngest child was not infected. These children shed infectious virus via the respiratory system, and this shedding observed in the nasopharynx cleared after five to 6 days; however, viral RNA was continuously detected in the children’s stool for more than four weeks [Wolf 2020]. Tang 2020 *et al.* reported an apparently asymptomatic (no fever or cough) 10-year-old child, from whom, 17 days after the last close contact with individuals testing positive for SARS-CoV-2, a fecal sample was positive for SARS-CoV-2 RNA. A retrospective study of 133 hospitalised COVID-19 patients identified 22 whose sputum or fecal samples tested positive after pharyngeal swabs became negative [Chen C 2020]. A study of 59 hospitalised COVID-19 patients reported that fecal discharge of SARS-CoV-2 RNA continued long after respiratory shedding had ceased [Cheung 2020]. Among fecal samples from 69 hospitalized COVID-19 patients, 20 tested PCR positive; SARS-CoV-2 RNA persisted for significantly longer in fecal samples than in oropharyngeal swabs [Wang X, Zheng J 2020].

***The role of aerosol-generating procedures in relation to orofecal transmission.*** One study reported the results of PCR tests among participants attending for upper GI endoscopy at a UK hospital [Hayee 2020]. Only individuals testing negative in nasopharyngeal swab PCR underwent their booked procedure. Post-procedure follow-up of 6,208 patients at one and two weeks identified no incident case of COVID-19.

### Viral culture

Viral culture using fecal samples was attempted in 6 studies [Jeong 2020, Kim JM 2020, Wang W, Xu Y 2020; Wölfel 2020; Xiao F 2020 and Zhang Y 2020], three reported culture of SARS-CoV-2 from stool samples [Wang W, Xu Y 2020; Xiao F 2020, Zhang Y 2020], and three did not [Jeong 2020, Kim JM 2020 and Wölfel 2020].

Wang W 2020 *et al.* reported two of four viral culture samples, however, no culture methods were reported; electron microscopy was performed suggesting the presence of the virus, but this observation does not show that the virus was viable. In Xiao F Sun J 2020, viral culture was attempted from an unreported number of specimens and cases and the cytopathic effect in Vero E cells was observed 2 days after a second-round passage. Zhang Y 2020 *et al.* reported isolating the virus from the stools of one severe hospitalised COVID-19 pneumonia case. The number of samples taken was unclear and while Vero cells were used for viral isolation from stool samples, culture methods were not described.

Additional evidence of SARS-CoV-2 replication activity was observed within the intestine: an inpatient for treatment of a rectal adenocarcinoma had samples taken from enteric sections, and the mucosa of rectum and ileum analysed [Qian 2020]. The rectal swab sample tested negative for SARS-CoV-2 RNA by PCR. However, typical coronavirus virions in rectal tissue were observed under electron microscopy with abundant lymphocytes and macrophages (some SARS-CoV-2 positive) infiltrating the lamina propria.

Methodological issues across these studies including variability in sample selection and methods of viral culture, reported in Table 5, mean the results may not be comparable and should be interpreted with caution.

***Studies of wastewater and sewage.*** 22 global studies investigated SARS-CoV-2 in wastewater or sewage, four looked at the role of toilets or sewage, and one reported observation in river water [Ahmed 2020, Ampuero 2020, Arora 2020, Chavarria-Miro 2020, Curtis 2020, Fongaro 2020, Fernandez-de-Mera 2020, Haramoto 2020, Hata 2020, Lara 2020, La Rosa 2020b, Medema 2020, Neault 2020, Ong 2020, Peccia 2020, Sharif 2020, Shutler 2020, Trottier 2020, Wang J 2020, Wang XW, Li J 2020, Wurtzer 2020, Zhao 2020].

All reported the detection of SARS-CoV-2 RNA and/or SARS-CoV-2 viral proteins; a number of studies suggested the potential of detection in sewage to be used as a public health monitoring system.

Wastewater surveillance may anticipate outbreaks: a Brazilian study identified SARS-CoV-2 RNA from sewage samples collected in November 2019 [Fongaro 2020]; a Spanish study detected sewage samples from 41 days prior to the declaration of the first COVID-19 case in Spain, and additionally in frozen samples dating back to 12 March 2019 [Chavarria-Miró 2020]. Virus concentrations found in wastewaters may correlate with prevalence in the local community. At very low community levels of circulation, sewage surveillance could detect SARS-CoV-2 RNA presence [La Rosa b 2020, Medema 2020]. 

### Viral culture in sewage

A study of hospital surfaces and sewage areas in China reported no viable virus was detected by culture of surface swabs or sewage samples [Wang J 2020]. Another study in China tested hospital sewage and found SARS-CoV-2 RNA but no viral culture of SARS-CoV was present in the sewage in their assays [Wang XW, Li J 2020].

***Toilets.*** An additional four studies investigated SARS-CoV-2 in toilets (Del Brutto 2020, Ding Z 2020, Kang 2020, Ong 2020]. A study in rural Guatemala reported the sole predictive variable was the use of the open latrines [Del Brutto 2020]. Ding 2020 *et al.* collected surface samples from a COVID-19 hospital in China reported toilet areas represented the most contaminated areas. Kang *et al.* reported the possibility of fecal aerosol spread but with little discussion of alternate pathways for transmission. They did not find any evidence of virus -laden bioaerosols. Ong and co-workers reported that 3 of 5 toilet sites (toilet bowl, sink, and door handle) in hospital settings were PCR-positive; anteroom and corridor samples were negative [Ong 2020].

***River water.*** One study tested river water for SARS-CoV-2: taking samples from three urban river locations in a low sanitation urban context (i.e. highly impacted by raw sewage) in Quito, Ecuador, during a peak of COVID-19 cases. SARS-CoV-2 RNA was detected in all samples, at levels similar to those in wastewater from cities during outbreaks [Guerreo-Latorre 2020]. Evidence from reviews can be found in Table 2.

**Table 2.** Included study characteristics: reviews.

Study (n= 33; 1 is a cohort and SR so appears in both 1a and 1b)	Fulfils systematic review methods	Research question (search date up to)	No. included studies (No. participants)	Main results	Key conclusions
Aboubakr 2020	no	To present the available data on the stability of coronaviruses (CoVs), including SARS‐CoV‐2, from previous reports to help understand its environmental survival (search date NR; study published June 2020)	Unclear (NA)	SARS‐CoV‐2 and other human and animal CoVs have short persistence on copper, latex and surfaces with low porosity vs other surfaces like stainless steel, plastics, glass & highly porous fabrics. SARS‐CoV‐2 is associated with diarrhoea & is shed in faeces. Some CoVs show persistence in human excrement, sewage and waters for a few days. Possible faecal–oral, foodborne & waterborne transmission of SARS‐CoV‐2 in regions using sewage‐polluted waters in irrigation & with poor water treatment systems.	Possible faecal–oral, foodborne & waterborne transmission of SARS‐CoV‐2 is a concern for regions using sewage‐polluted waters in irrigation & with poor water treatment systems.
Achak M 2020	no	To review evidence on the presence of SARS-CoV-2 in aqueous environments, and to describe the knowledge on detection and survival of SARS-CoV-2 in wastewater and hospital wastewater, in order to understand the different routes of SARS-CoV-2 transmission (search date NR; study available online 24 October 2020).	NR	SARS-CoV-2 RNA is identified in a range of water environments including hospital wastewaters. According to a few studies investigating the deactivation of SARS-Co V-2 showed that chlorine- based disinfectants are widely used for their broad sterilization spectrum, high inactivation efficiency and easy decomposition with little residue, as well as represents the best economic solution. The complete deactivation of SARS-CoV-2 can be achieved by combination of other technologies (biological and/or physical-chemical processes).	Authors suggest there is a need to develop more secure, efficient, economical disinfection technologies in order to limit the transmission of Covid-19 and to avoid other waves of the pandemic of Covid-19 infections
Aguiar-Oliveira M 2020	no	To summarize the current global experience on SARS-CoV-2 wastewater based epidemiology in distinct continents and viral detection in polluted surface water. The advantages and concerns of this strategy for SARS-CoV-2 surveillance are discussed. (Search date NR; article published 10 December 2020).	NR	Viral RNA has been detected in the stool of Covid-19 patients and viable viruses had been isolated in some of these samples. Thus, a putative role of SARS-CoV-2 fecal-oral transmission has been argued. SARS-CoV- 2 is shed in human excreta and further disposed in the sewerage or in the environment, in poor basic sanitation settings.	Outcomes suggest that wastewater based epidemiology is a valuable early warning alert and a helpful complementary surveillance tool to subside public health response, to tailor containment and mitigation measures and to determine target populations for testing. In poor sanitation settings, contaminated rivers could be alternatively used as a source for environmental surveillance.
Amirian 2020	no	Current knowledge on the potential for fecal transmission is briefly reviewed and the possible implications are discussed from a public health perspective (search date NR; study published April 2020)	Unclear (NR)	A number of case series have reported the presence of SARS-CoV-2 genetic material in the stool of some COVID-19 patients.	If orofecal transmission is established, it will impact public health guidelines on sewage exposure, nosocomial infections, residential care facilities, and food preparation.
Cahill 2020	no	Are recreational waters a potential transmission route for SARS-CoV-2 to humans? (search date NR; study published October 2020)	Unclear (NR)	SARS-CoV-2 has been detected in faeces and wastewater in recent months. Wastewater is a potential dissemination route for SARS-CoV-2 to recreational waters. Limited data on the presence and viability of SARS-CoV-2 in water bodies exists.	The risk of SARS-CoV-2 exposure to recreational water users is believed to be low. Further research is required.
Cheung K 2020	no	To review the evidence on gastrointestinal symptoms and detection of virus in stool in Covid-19. (11 March 2020)	60 (4,243)	Pooled prevalence of all GI symptoms was 17.6% (95%CI 12.3 to 24.5); 11.8% of patients with nonsevere COVID-19 had GI symptoms (95%CI 4.1 to 29.1), and 17.1% of patients with severe COVID-19 had GI symptoms (95% CI, 6.9 to 36.7). Pooled prevalence of stool samples positive for virus RNA was 48.1% (95% =CI 38.3 to 57.9); of these samples, 70.3% of those collected after loss of virus from respiratory specimens tested positive (95% CI 49.6 to 85.1).	Around 18% of Covid-19 patients experienced GI symptoms. Viral RNA can be identified in stool samples of around half of Covid-19 patients, and is often detectable even after respiratory samples test negative.
Chiappini 2020	no	To give an overview of gastrointestinal involvement in children with SARS‐ COV‐2 infection. (Search date NR; article published 24 November 2020)	NR	Diarrhea and vomiting have been reported in about 8%‐9% of cases, reaching more than 20% in some studies. Fecal shedding in children has been reported in 20%‐30% of children and has been observed in both those with and those without overt GI involvement.	GI symptoms are common findings in children with SARS‐CoV‐2 infection. Fecal shedding in asymptomatic children and prolonged fecal elimination, lasting several days after negativization of RT‐PCR on respiratory swabs, have been reported with variable frequency in children with Covid‐19.
Collivignarelli 2020	no	To review the evidence on the presence of SARS-CoV-2 in wastewater and sewage sludge, the factors affecting its inactivation and the main proposed treatments. (search date NR; preprint posted June 2020).	20 (NA)	Literature on SARS-CoV-2 in wastewaters is currently limited. For SARS-CoV-1, its resistance in wastewater is limited, especially at temperatures above 20 °C, and the virus has been easily removed with chlorine (> 0.5 mg L-1 for 30 min).	Detection of SARS-CoV-2 in wastewater might track the epidemic trends: although promising, an effective and wide application of this approach requires a deeper knowledge of the amounts of viruses excreted in faeces, and the actual detectability of viral RNA in sewage.
Cuicchi D 2020	no	To collect the data available on SARS- CoV-2 in the GI system and evaluate whether the digestive system could contribute to viral transmission (31 May 2020).	27 (671)	46.5% patients had a positive stool sample for SARS- CoV-2 RNA; 63.9% remained positive after pharyngeal swabs became negative; in 3 studies, in samples of 3 patients out of 8 examined, SARS-CoV-2 RNA was found in GI tissues. Live virus in stool samples was confirmed in two studies and not found in another study. These results suggested that SARS-CoV-2 could infect gastrointestinal epithelial cells and it may be transmitted through the digestive tract.	These results suggested that SARS-CoV-2 could infect GI epithelial cells and it may be transmitted through the digestive tract.
Ding S 2020	no	Is SARS-CoV-2 also an enteric pathogen with potential fecal-oral transmission? (search date NR; review completed 23 April 2020)	NR	SARS-CoV-2 is capable of infecting the gastrointestinal tract and shedding in the environment for potential human-to-human transmission; more reserach in needed to understand the extent and relative importance of these. .	Work is needed to examine the full extent of GI and liver aspects of COVID-19
Dona 2020	no	To review evidence on GI symptoms and the use of rectal swabs to detect SARS-CoV-2 RNA, in children. (search date NR; study published July 2020).	NR	GI symptoms have been reported more commonly among children than adults with Covid-19. SARS-CoV- 2 RNA has been detected in several studies of rectal swabs in children.	Further evidence on GI involvement and excretion of SARS-CoV-2 in faeces is needed to confirm fecal viral loads regardless of enteric symptoms, and to better explore viral RNA detection across the disease course.
Edwards 2020	yes	To describe whether SARS-CoV-2 viral loads (VLs) and cycle thresholds (CTs) vary by sample type, disease severity and symptoms duration (8 April 2020)	24 (173)	Viral loads higher in saliva and sputum vs NP swabs; in asymptomatics; & in severe COVID-19. Stool samples positive for a longer period than other samples.	Diagnostic strategies should consider these variations in viral loads.
El-Wahab EWA 2020	no	Summarize the ways in which SARS- CoV-2 is transmitted and provide scientific support for the prevention and control of COVID-19 (31 July 2020).	302 (NR)	The basic mechanisms of SARS-CoV-2 transmission person-to-person contact through respiratory droplets, or via indirect contact... Although SARS-CoV-2 has been detected in non-respiratory specimens, including stool, blood and breast milk, their role in transmission remains uncertain.	Additional research is needed to better understand transmission.
Gupta 2020	no	To establish the incidence and timing of positive faecal samples for SARS‐ CoV‐2 in patients with Covid‐19 (3 April 2020)	26 (NR)	Persistent RNA shedding has been recorded in a number of studies, even subsequent to respiratory test negativity. Notably, in one study, 44/ 153 stool specimens tested were PCR positive and live virus was detected in 2 of 4 specimens culture.	There is a high incidence and persistence of positive faecal RT‐PCR tests for SARS‐CoV‐2 after negative nasopharyngeal swabs in patients with Covid‐19.
Han Z 2020	no	To review published studies about discharged patients testing positive again for SARS‐ CoV‐2 RNA. (27 April 2020)	12 (90)	All studies were in China. These reports indicates the presence of discharged patients who remain asymptomatic but test PCR- positive; however, it is unclear whether they are contagious	This review suggests the need for parallel testing of different samples e.g. fecal specimens, from Covid‐19 patients before and after they are discharged from hospitals.
Ji B 2020	no	To provide a comprehensive profile on the transmission characteristics of the coronaviruses in water, sludge, and air environment, especially the water and wastewater treatment systems. (Search date NR; paper available online 27 October 2020).	NR	Existing drinking water treatment protocols effectively remove SARS-CoV-2 and to date, drinking water is safe. SARS-CoV-2 is shed in the faeces and urine of infected individuals and then enters wastewater. Wastewater monitoring could be a strategic surveillance tool.	Intensive studies of SARS-CoV-2 activity and survival in water and wastewater treatment are highly desirable.
Jones 2020	no	To critically evaluate the incidence of GI symptoms, the quantity and infectivity of SARS-CoV-2 in feces and urine, and whether these pose an infection risk in sanitary settings, sewage networks, wastewater treatment plants, and the wider environment e.g. rivers, lakes and marine waters. (search date NR; study published 20 December 2020)	48 (NA)	SARS-CoV-2 RNA can be readily detected in feces and occasionally urine. Severe GI dysfunction only occurs in a small number of cases (11 ± 2%). Likelihood of SARS-CoV-2 being transmitted via feces appears very low. Likelihood of infection from sewage-contaminated water or food is extremely low.	The likelihood of SARS-CoV-2 being transmitted via feces or urine appears low due to the low relative amounts of virus present in feces/urine.
Karia 2020	yes	To review the sources of viral shedding that have been reported to date and compare the duration of shedding from different sources and their relation to clinical recovery (July 2020)	19 (1,433)	Prolonged shedding observed in a range of specimens; max duration to conversion 60 days; in several studies, viral shedding from the GI tract was for a longer duration and at a greater viral load than from the respiratory tract.	Prolonged viral shedding is important to consider while discontinuing isolation procedures and/or discharging SARS-CoV-2 patients.
Kingsbury 2020	no	What is international best practice regarding reducing the likelihood that food products or packaging are vectors for COVID-19? In this context, sources of COVID-19 may be production or supply chain workers? What is international best practice for mitigation options to reduce transfer of COVID-19 from workers to food products? (28 April 2020)	> 77 (NR)	Infectious virus has been found in faeces of some infected people, raising the possibility of faecal-oral transmission via contaminated vehicles such as food, but there is no evidence for this having occurred.	Best practice for reducing the risk of contamination of food products or packaging continues to be managing the risk of SARS- CoV-2 infection amongst workers.
La Rosa a	no	Scoping review (23 February 2020)	12 (N/A)	Coronaviruses seems to have a low stability in the environment and be very sensitive to oxidants such as chlorine; coronaviruses appear to be inactivated significantly faster in water than non-enveloped human enteric viruses with known waterborne transmission; temperature is an important factor influencing viral survival: the titer of infectious virus declines more rapidly at 23 to 25 °C than at 4 °C; there is not current evidence that human coronaviruses are present in surface or ground waters or are transmitted through contaminated drinking-water.	Coroviridae have been isolated in different types of liquids from waste to surface water, but in general they appear to be unstable. Chlorination and higher temperatures lead to their inactivation. At the time of search (Feb 2020) there was not evidence of coronavirus transmission through contaminated water.
Mehraeen 2020	no	To identify current evidence on transmission modes of Covid-19. (April 2020).	36 (NR)	The review identified five potential transmission modes of COVID-19 including airborne, droplet, contact with contaminated surfaces, oral and fecal secretions. Furthermore, some studies have pointed out other modes of virus transmission, such as person to person, and direct contact with animals.	Droplet and contact with contaminated surfaces were the most frequent transmission modes of COVID-19. Fecal excretion, environmental contamination, and fluid pollution might contribute to a viral transmission. The possibility of fecal transmission of Covid-19 has implications, especially in areas with poor sanitation.
Meyerowitz 2020	no	To review the evidence on transmission of SARS-CoV-2 (7 September 2020)	NR	Strong evidence from case and cluster reports indicates that respiratory transmission is dominant, with proximity and ventilation being key determinants of transmission risk. Although live virus has been isolated from saliva and stool and viral RNA has been isolated from semen and blood donations, there are no reported cases of SARS-CoV-2 transmission via fecal–oral, sexual, or bloodborne routes. To date, there is 1 cluster of possible fecal–respiratory transmission.	Evidence indicates that respiratory transmission is dominant. Re: fecal aerosol transmission, given how rarely live virus has been isolated from stool, the low levels of replication-competent virus in stool that might be aerosolized from toilet flushing seem highly unlikely to cause infection except under unusual or extraordinary circumstances.
Mohapatra S 2020	no	To review evidence on the detection, occurrence and fate of SARS-CoV-2 (& other enveloped viruses) during primary, secondary, and tertiary wastewater and water treatment processes. (Search date NR; article available online 6 October 2020).	NR	SARS-CoV-2 contamination of water bodies may be possible through orofecal route. SARS-CoV-2 RNA has been detected in wastewater across the globe. Coagulation-flocculation, filtration can remove SARS- CoV-2 RNA and complete inactivation of SARS-CoV-2 is possible through chlorination.	More research on the possibility of faecal-oral transmission and its possible fate and persistence in various environmental compartments is needed.
Morone 2020	yes	To correlate the presence and the relevant temporal patterns of SARS-CoV-2 viral RNA in biological specimens (stool, urine, blood, and tears) of the transmission with clinical/ epidemiological features in patients with Covid-19 (5 May 2020)	55 (1,348)	Fecal positivity duration (median 19 days) was significantly (p < 0.001) longer than respiratory tract positivity (median 14 days). Limited data are available about the other specimens.	Attention should be paid to negativization criteria for COVID- 19, because patients could have longer alternative viral shedding.
Pamplona 2020	yes	To describe the evidence on GI symptoms, enteric involvement and fecal excretion of SARS-CoV-2 viral RNA, and to discuss the possible fecal- oral transmission pathway of Covid-19. (12 April 2020)	33 (NR)	High variabilty in GI symptom reporting, summary estimate GI symptoms present in 16%, diarrhea in 8.1%, nausea-vomiting in 12% and abdominal pain in 4% of Covid-19 patients.	GI symptoms are common in SARS CoV-2 infection at the time of patient admission, sometimes preceding respiratory symptoms, and sometimes represent the only clinical manifestation.
Parasa 2020	yes	What are the incidence rates of gastrointestinal symptoms among patients with SARS-CoV-2 infection? (30 March 2020)	29 (4805)	Pooled rates were 7.4% (95%CI 4.3% to 12.2%) of patients reporting diarrhea and 4.6% (95%CI 2.6% to 8.0%) of patients reporting nausea or vomiting. Fecal tests that were positive for SARS-CoV-2 were reported in 8 studies, and viral RNA shedding was detected in feces in 40.5% (95% CI, 27.4%-55.1%) of patients. There was high level of heterogeneity (I2 = 94%), but no statistically significant publication bias noted.	These findings suggest that that 12% of patients with COVID-19 will manifest GI symptoms; however, SAR-CoV-2 shedding was observed in 40.5% of patients with confirmed SARS-CoV-2 infection.
Patel 2020	no	To update the current literature on transmission of SARS-CoV-2. (search date NR; study published 7 July 2020)	NR	The rationale behind its transmission potential is that viral RNA has unexpectedly been detected in multiple bodily fluids, with some samples having remained positive for extended periods of time. Additionally, the receptor by which the virus gains cellular entry, ACE2, has been found to be expressed in different human body systems, thereby potentiating its infection in those locations.	Detection of viral RNA shedding in multiple bodily fluids/samples suggests the potential for modes of transmission additional to respiratory, such as bloodborne, urinary, and fecal-respiratory. Transmission by such means remains controversial given the limited supporting data for each mode.
SAGE 2020	no	To describe SARS-CoV-2 transmission routes and environments (search date NR; report available online 22 October 2020).	NR	Orofecal-related results: SARS-CoV-2 RNA has been found in stool samples and RNA shedding often persists for longer than in respiratory samples; however, isolation of live virus has rarely been successful from stool or urine. The GI tract probably is also susceptible to infection and may serve as a transmission portal given the high concentration of ACE2 receptors in the small bowel, however, no published reports have described faecal-oral transmission. Positive RNA samples have been found in samples. There is only indirect evidence of orofecal transmission in this pandemic.	More evidence is needed to understand transmission. Research should be embedded into the public health responses to the pandemic.
Santos 2020	yes	To investigate differences in viral shedding in respiratory and fecal samples from children with Covid-19. (19 April 2020)	4 (36)	A higher proportion of children had viral shedding in stools after 14 days of symptoms onset compared to respiratory samples (RR= 3.2, 95%CI 1.2 to 8.9, I2 = 51%). Viral RNA shedding was longer in fecal samples with a mean difference of approximately 9 days (Mean Difference = 8.6, 95%CI 1.7 to 15.4, I2 = 77%) compared with respiratory samples	SARS-CoV-2 shedding seems to be present in feces for a longer time than in the respiratory tract of children. Although fecal SARS-CoV-2 presence in feces do not confirm its transmissibility, the high and fast spread of the COVID-19 disease worldwide indicate other transmission routes are also plausible
Sehmi 2020	no	To establish if there is any evidence of the presence of live SARS-CoV-2 in feces of Covid-19 patients.n (6 June 2020)	4 (8)	1 study successfully isolated live SARS-CoV-2 virus in the stool sample of a patient with severe Covid-19, using Vero cell culture & electron microscopy. Another study using similar methods isolated and demonstrated live virus in 2 out of 3 patients who had tested PCR positive in stool samples. The same authors in another report mentioned their unpublished findings on successful isolation of infectious SARS-CoV-2 from stool. In a fourth study, ientification of live virus in stool was attempted successfully in 2 out of 4 patients; neither of these patients had GI symptoms.	Live SARS-CoV-2 virus is present in fecal samples of Covid-19 patients, and therefore supports the hypothesis that Covid-19 could potentially be transmitted via the feco-oral route.
Sun S 2020	no	To review fecal transmission of Covid-19, the practices of open defecation, and the resultant routes of transmission of fecal pathogens. Also, we highlight the open design of common squat toilets and the potential exposure to fecal droplets and residues. (Search date NR; article published 29 November 2020).	NR	In communities practicing open defecation, poor hand hygiene, contaminated shoes and objects, mechanical vectors, and outdoor human activities could all contribute to fecal transmission. Other risk factors include squat pans with lidless designs and open flushing mechanisms, in-cubicle open waste bins, and the lack of water-sealing U-traps in squat toilets.	Fecal-associated transmission has been identified as a potential route of virus spread in the Covid-19 pandemic, as an increasing body of evidence confirmed high viral loadings and infectivity of SARS-CoV-2 in patients’ stools, including asymptomatic, pre-symptomatic, and convalescent individuals.
Tian 2020	no	To report on the gastrointestinal manifestations and pathological findings of patients with COVID‐19, and to discuss the possibility of faecal transmission. (search date NR; study published 31 March 2020)	NR (2023 patients)	With an incidence of 3 to 79%, GI symptoms included anorexia 40 to 50%, diarrhoea 2% to 50%, vomiting 4 to 67%, nausea 1 to 29%, abdominal pain 2 to 6% and GI bleeding 4 to 14%. Diarrhoea was observed before and after diagnosis with a mean duration of 4.1 ± 2.5 days. Adult and children patients can present with digestive symptoms in the absence of respiratory symptoms.	
Tran 2020	no	To review evidence on the presence of SARS-CoV-2 in water and wastewater and what level of risk it may pose. (search date NR; study published 1 October 2020)	NR	SARS-CoV-2 has been detected in water and wastewater. The suggested transmission route of SARS- CoV-2 into water through stool and mask of infected patients. Coronavirus is often inactivated rapidly in water. Paper-based devices have been suggested for detecting traces of SARS-CoV-2 in water. Existing disinfection processes possibly sufficient to kill SARS- CoV-2 in water.	The presence of SARS-CoV-2 RNA in river water and untreated wastewater is confirmed, but strong evidence of its survival time in water environments is missing. One study confirmed lack of infectivity of SARS-CoV-2 in water based on absence of cytopathic effect.
van Doorn 2020	no	To critically assess the clinical relevance of testing stool samples and anal swabs and provide an overview of the potential faecal‐oral transmission of SARS‐CoV‐2 (7 July 2020)	95 (2,149)	934/2149 (43%) patients tested positive for SARS‐CoV‐2 in stool samples or anal swabs, with positive test results up to 70 days after symptom onset. A meta‐analysis executed with studies of at least 10 patients revealed a pooled positive proportion of 51.8% (95% CI 43.8 ‐ 59.7%). Positive faecal samples of 282/443 patients (64%) remained positive for SARS‐CoV‐2 for a mean of 12.5 days, up to 33 days maximum, after respiratory samples became negative for SARS‐CoV‐2. Viable SARS‐CoV‐2 was found in 6/17 (35%) patients in whom this was specifically investigated.	Viral shedding of SARS‐CoV‐2 in stool samples occurs in a substantial proportion of patients, making faecal‐oral transmission plausible. Furthermore, detection in stool samples or anal swabs can persist long after negative respiratory testing.
Zhen-Dong 2020	no	To assess epidemioogy and clinical features of Covid-19 among Chinese children.	37 (406)	Asymptomatic infections and mild cases account for 44.8%, with only 7 cases of critical illness; laboratory examination of lymphocyte counts is not reduced, as it is for adults; chest CT findings are less severe than those for adults. These presentations are the clinical features of COVID-19 in children. Only 55 of the 406 cases were tested by anal swab for virus nucleic acid, 45 of which were positive, accounting for 81.8% of stool samples.	There are more children than adults with asymptomatic infections, milder conditions, faster recovery, and a better prognosis. Some concealed morbidity characteristics also bring difficulties to the early identification, prevention and control of COVID-19.

**Table 3.** Quality of included primary studies.

Study	Study type	Description of methods with sufficient detail to replicate	Sample sources clear	Analysis & reporting appropriate	Is bias dealt with	Applicability
Agrawal 2020	Observational	Yes	Yes	Yes	Unclear	Yes
Ahmed 2020	Observational	Yes	No	Yes	No	Yes
Ampuero 2020	Observational	Yes	Yes	Yes	No	Yes
Arora 2020	Observational	Yes	Yes	Yes	Unclear	Yes
Betancourt 2020	Observational	Yes	Yes	Yes	Unclear	Yes
Chan 2020	Observational	Yes	Yes	Unclear	No	Unclear
Chavarria-Miro 2020	Observational	Yes	No	Yes	Unclear	Unclear
Chen C 2020	Observational	Yes	Yes	Not Applicable	Not Applicable	Unclear
Chen W 2020	Observational	Yes	Unclear	Yes	Unclear	Yes
Chen Y 2020	Observational	Yes	Yes	No	No	No
Cheung CCL 2020	Observational	Yes	Yes	Yes	Not Applicable	Yes
Cheung K 2020	Observational	Yes	Yes	Yes	Unclear	Yes
Cho 2020	Observational	Yes	Yes	Yes	No	Yes
Chu H 2020	Observational	Yes	Yes	Yes	Not Applicable	Yes
COVID Ix Team	Observational	Yes	Yes	Yes	Unclear	No
Curtis 2020	Observational	Yes	Yes	Yes	Unclear	Unclear
Del Brutto 2020	Observational	Yes	Yes	Yes	Unclear	Yes
Ding Z 2020	Observational	Yes	Not Applicable	Yes	Unclear	Yes
Fernández‐de‐Mera 2020	Observational	Yes	Yes	Unclear	No	Unclear
Fongaro 2020	Observational	Yes	Yes	Yes	Unclear	Yes
Ge 2000	Observational	Yes	Yes	Unclear	Not Applicable	Yes
Guerrero-Latorre 2020	Observational	Yes	Yes	Yes	Unclear	Yes
Han C 2020	Observational	Yes	No	Yes	No	No
Haramoto 2020	Observational	Yes	Yes	Yes	Not Applicable	Yes
Hata 2020	Observational	Yes	Yes	Yes	Not Applicable	Yes
Hayee 2020	Observational	Yes	Not Applicable	Yes	Unclear	Yes
Hoehl M 2020	Observational	Yes	Yes	Yes	Unclear	Yes
Holshue 2020	Observational	Yes	Yes	Yes	Unclear	No
Iglesias 2020	Observational	Yes	Yes	Yes	Unclear	Yes
Izquierdo-Lara 2020	Observational	Unclear	Yes	Yes	Unclear	Yes
Jeong 2020	Observational	Yes	Yes	Yes	Unclear	Yes
Jiehao 2020	Observational	Yes	Yes	Yes	Unclear	No
Kang 2020	Observational	Yes	Yes	Yes	Unclear	Yes
Kim J-M 2020	Observational	Yes	No	Unclear	No	Unclear
La Rosa 2020 (b)	Observational	Yes	Unclear	Yes	Not Applicable	Not Applicable
Lescure 2020	Observational	Yes	Yes	Yes	Unclear	No
Li 2020	Observational	Unclear	Yes	Yes	Unclear	Unclear
Ling 2020	Observational	Yes	Unclear	Yes	Unclear	No
Lo 2020	Observational	Yes	Yes	Unclear	Unclear	No
Medema 2020	Observational	Yes	No	Yes	No	Yes
Neault 2020	Observational	Yes	Unclear	Yes	Not Applicable	Yes
Nicastri 2020	Observational	Yes	Yes	Yes	Unclear	No
Ong 2020	Observational	Yes	No	Unclear	Unclear	Yes
Pan 2020	Observational	Unclear	No	Yes	Unclear	No
Peccia 2020	Observational	Unclear	Yes	Yes	Not Applicable	Yes
Peng 2020	Observational	No	Yes	Not Applicable	No	No
Qian 2020	Observational	Yes	Yes	Yes	Unclear	Yes
Senapati 2020	Observational	Unclear	Yes	Not Applicable	Not Applicable	No
Sharif 2020	Observational	Yes	Yes	Yes	Unclear	Yes
Shutler 2020	Observational	Yes	No	No	Unclear	No
Tan 2020	Observational	Unclear	Yes	Yes	Unclear	No
Tang 2020	Observational	Unclear	Unclear	Yes	Unclear	No
Trottier 2020	Observational	Yes	Yes	Yes	Not Applicable	Yes
Wang J 2020b	Observational	Yes	No	Yes	No	Yes
Wang Q-X 2020	Observational	Yes	Yes	Yes	No	Unclear
Wang S 2020	Observational	Unclear	Yes	Yes	No	Unclear
Wang W 2020	Observational	Unclear	Yes	Unclear	Unclear	Unclear
Wang X, Zhou Y 2020	Observational	Yes	Yes	Unclear	Unclear	No
Wang X, Zheng J 2020	Observational	Yes	Yes	Yes	Unclear	Yes
Wolf 2020	Observational	Yes	Yes	Yes	Unclear	Yes
Wölfel 2020	Observational	Unclear	Unclear	Unclear	Yes	Unclear
Wu Y 2020	Observational	Yes	No	Yes	No	No
Wurtzer 2020	Observational	Yes	No	Yes	Unclear	Yes
Xiao F & Tang M 2020	Observational	Yes	Yes	Not Applicable	No	Unclear
Xiao F & Sun J 2020	Observational	Yes	No	Not Applicable	Not Applicable	Unclear
Xing Y 2020	Observational	Yes	Unclear	Unclear	Unclear	Yes
Xu Y 2020	Observational	Yes	Yes	Not Applicable	Not Applicable	Unclear
Yang Y 2020	Observational	Yes	Yes	Yes	Unclear	Yes
Young 2020	Observational	Unclear	Unclear	Yes	Unclear	No
Yuan 2020	Observational	Unclear	Yes	Yes	Unclear	No
Zhang J 2020	Observational	Yes	Unclear	Unclear	No	Unclear
Zhang T 2020	Observational	Yes	Yes	Yes	Unclear	Yes
Zhang W 2020	Observational	Yes	Yes	Unclear	Unclear	Unclear
Zhang Y 2020	Observational	Unclear	No	Yes	Unclear	No
Zhang Z, Chen C 2020	Observational	No	Yes	Yes	Unclear	Yes
Zhao 2020	Observational	Yes	Yes	Yes	Not Applicable	Yes

**Table 4.** Main findings of included primary studies: SARS-CoV-2 and the role of orofecal transmission (n=76).

Study	Main findings of primary studies on orofecal transmission of SARS-CoV-2
Chan 2020	This very early study established the likelihood of person to person transmission of SARS-CoV-2, in hospital and family settings. The two faecal samples from patients 3 and 4 who had preceding diarrhoea were negative on a multiplex PCR assay for common diarrhoeal viruses, bacteria, and parasites.
Chen C 2020	This retrospective study of 133 hospitalised COVID-19 patients identified 22 whose sputum or fecal samples tested positive, after their pharyngeal swabs became negative.
Chen W 2020	SARS-CoV-2 RNA was readily detected in the blood (6/57 patients) and anal swabs (11/28 patients).
Chen Y 2020	Sixty seven percent (28/42) laboratory-confirmed hospitalised COVID-19 patients tested positive for SARS-CoV-2 RNA in stool specimens; this was not associated with the presence of GI symptoms or severity of illness. Among them, 18 (64%) patients remained positive for viral RNA in the feces after the pharyngeal swabs turned negative, for a duration of 6 to 10 days.
Cheung CCL 2020	This study used multiplex immunohistochemistry and unexpectedly detected SARS-CoV-2 viral antigens in intestinal and liver tissues, in surgical samples obtained from two hospitalized patients who recovered from Covid-19. The presence of the virus was validated by RT-PCR and flow cytometry to detect SARS- CoV-2-specific immunity in the tissues.
Cheung K 2020	This study analysed stool samples from a cohort of 59 patients with COVID-19 in Hong Kong during February 2020 and additionally did a meta-analysis of data from 11 studies on the prevalence of GI symptoms and stool excretion of viruses. Fecal discharge continues long after respiratory shedding of COVID-19 has ceased.
Cho 2020	This case study reports an infant with mild Covid-19, positive-to-negative nasal swab conversion occurred on the 21st day from the onset of symptoms, but stool swab positivity persisted during the 6-week admission period and for 7 weeks during follow-up at an outpatient clinic after discharge.
Chu H 2020	Case report of a breastfeeding woman with a positive PCR test for SARS-CoV-2. The patient presented on 24 January 2020 with GI symptoms; later she developed a fever. Her infant had been born 16 January 2020. She tested PCR positive in respiratory swabs. She had persistent SARS-CoV-2 RNA positivity in her feces but negativity in her breastmilk. She bottle fed her baby with her breastmilk after treatment. The baby appeared healthy and unaffected after a 1-month follow up.
COVID Ix Team	SARS-CoV-2 RNA was detected in at least one nasopharyngeal (NP) swab, 11/12 oropharyngeal (OP) swab and 7/10 in the stool in this case series describing the first 12 US patients confirmed to have COVID-19 from 20 January to 5 February 2020.
Ge 2020	This case study of a hospitalised Covid-19 patient reported that the fecal samples remained PCR-positive for 22 days after their respiratory samples turned negative.
Han C 2020	Among a group of hospitalised patients with low severity COVID-19, digestive symptoms were present in 57%. Patients with digestive symptoms were more likely to be fecal virus-positive than those with respiratory symptoms.
Hayee 2020	This study reports PCR test results for outpatients attending for GI endoscopy at a UK hospital 30th April to 30th June 2020: 3/2,611 asymptomatic patients tested positive for SARS-CoV-2 on nasopharyngeal swab testing pre-endoscopy. No cases of Covid-19 were detected for 14 days after the procedure.
Hoehl S 2020	Children and staff at 50 day care centres in Germany were tested repeatedly over 12 weeks. Buccal mucosal swabs and anal swabs were taken (by parents) from 825 children aged 3 months to 8 years attending the day care centres and 372 staff members (swabs self-collected) of these settings, between 18 June and 10 September 2020. 7,366 buccal mucosa swabs and 5,907 anal swabs were analysed. No respiratory or GI shedding of SARS-CoV-2 was detected in any of the children. Two adult staff members at two different day care centers tested positive; one had symptoms.
Holshue 2020	Stool obtained from a single hospitalized Covid-19 case was positive for SARs-CO-V-2 on day 7 of the illness.
Jeong 2020	There was viable SARS-CoV-2 in saliva, urine, and stool from COVID-19 patients up until days 11 to 15 of the clinical course suggesting that viable SARS-CoV-2 can be secreted in various clinical samples as well as respiratory specimens.
Jiehao 2020	Prolonged virus shedding was observed in the respiratory tract and feces of children at the convalescent stage.
Kim J-M 2020	SARS-CoV-2 RNA was detected in serum, urine or stool samples in 20% of patients hospitalised with Covid-19: 13/129 stool samples obtained from 74 patients tested positive. However, the virus could not be isolated from these samples and therefore the risk of transmission via these media is not established.
Lescure 2020	A case series of the first five identified Covid-19 cases in Europe; an early demonstration of the vastly increased risk for elderly versus younger people. Viral RNA was detected in the stools of the two paucisymptomatic women.
Li 2020	This case series reported on 29 hospitalised mild-to-moderate severity Covid-19 patients in China. Fecal samples from 4 patients tested positive for SARS-CoV-2 RNA, including one apparently asymptomatic case.
Ling 2020	From 292 confirmed cases with COVID-19 in the Shanghai region, 66 recovered patients were included. Clearance of viral RNA in patients’ stools was delayed compared to oropharyngeal swabs.
Lo 2020	A report of the clinical and microbiological features of ten hospitalized Covid-19 patients in Brazil between 21 January and 16 February 2020 found that SARS- CoV-2 can be shed in the stool.
Nicastri 2020	SARS-CoV-2 RNA was positive in stools, nasopharyngeal and oropharyngeal swabs at different time points in a case report.
Pan 2020	Stool samples from 9/17 confirmed patients were positive on RT-PCR analysis.
Peng 2020	Virus was found in urine, blood and in two anal swabs and oropharyngeal swabs of nine patients diagnosed with COVID-19.
Qian 2020	SARS-CoV-2 was detected in the rectum of a COVID-19 patient during the disease incubation period. There was direct evidence of replication of SARS-CoV-2 in the intestine.
Senapati 2020	This pilot study in India found SARS-CoV-2 RNA in fecal samples from 12 symptomatic and asymptomatic COVID-19 patients.
Tan 2020	In a single case report, SARs-CoV-2 was detected in the throat and rectum of the patient with COVID‐19.
Tang 2020	An asymptomatic child was positive for a coronavirus by reverse transcription PCR in a stool specimen 17 days after the last virus exposure. The child was virus positive in stool specimens for at least an additional 9 days.
Wang Q-X 2020	Case series of 5 individuals who had Covid-19 and whose respiratory samples were negative by PCR, but had positive fecal samples. Observed over 3 to 15 days, no cases of reinfection occurred and all fecal samples became negative..
Wang S 2020	Retrospective case series using clinical records, laboratory results, and CT findings for 17 COVID-19 patients including fecal sample testing showed 11/17 had positive PCR tests in fecal samples.
Wang W, Xu Y 2020	In this case series from China, 2 stool specimens out of 44 positives contained live virus, suggesting that orofecal transmission is possible. Transmission of the virus by respiratory and extra respiratory routes may help explain the rapid spread of disease.
Wang X, Zheng J 2020	Among fecal samples from 69 hospitalized Covid-19 patients, 20 tested PCR positive. The duration of SARS-CoV-2 RNA persistence was significantly longer in fecal samples than in oropharyngeal swabs.
Wang X, Zhou Y 2020	In three hospitalised cases, intestinal SARS-CoV-2 infection affected the disease course of Covid-19 and stool samples were positive for SARS-CoV-2 by RT-PCR.
Wolf 2020	A five person family with a confirmed Covid-19 case was hospitalized and observed: the parents and 2 children aged 2 and 5 years became infected but the youngest child was not infected. These children shed infectious virus via the respiratory system, and this shedding observed in the nasopharynx cleared after 5 to 6 days; however, viral RNA was continuously detected in the children’s stool for more than 4 weeks
Wölfel 2020	A detailed virological analysis of 9 Covid-19 hospitalised patients that provide proof of active virus replication in tissues of the upper respiratory tract. Stool samples were also positive for RNA, and in one case the course of RNA concentration in stools indicated replication in the GI tract. However, attempts to culture live virus from fecal samples were unsuccessful in 0/13 samples (from 4 patients).
Wu Y 2020	In 98 hospitalized Covid-19 cases, patients’ faecal samples remained positive for SARS-CoV-2 for a mean of 11 days (maximum 5 weeks) after respiratory tract samples became negative.
Xiao F, Tang M 2020	39 of 73 hospitalized Covid-19 patients aged 10 months to 78 years tested positive for SARS-CoV-2 in fecal samples. Gastric, duodenal and rectal epithelial specimens collected via endoscopy of one patient were also studied: Immunofluorescence data showed that ACE2 protein, proven to be a cell receptor for SARS-CoV-2, is abundantly expressed in the glandular cells of gastric, duodenal, and rectal epithelia, supporting entry of SARS-CoV-2 into the host cells. Intracellular staining of viral nucleocapsid protein in gastric, duodenal, and rectal epithelia showed that SARS-CoV-2 infects these GI glandular epithelial cells. Viral RNA was also detected in esophageal mucous tissue, but the absence of viral nucleocapsid protein staining in esophageal mucosa indicates low viral infection in esophageal mucosa. Detection of some viral nucleocapsid protein in rectal epithelial cells was observed in some additional Covid-19 patients, suggesting that some infectious viral particles may survive the GI environment.
Xiao F, Sun J 2020	This case series of 28 hospitalised patients for whom feces samples were available indicated that infectious virus was present in feces from two cases who also tested positive for viral RNA by RT-PCR.
Xing Y 2020	Three children showed a prolonged presence of SARS‐CoV‐2 in feces after throat swabs were negative.
Xu Y 2020	This study of 10 children with COVID-19 found that symptoms among children were nonspecific and relatively mild; rectal swabs tested positive among 8/10 cases even once nasopharyngeal tests became negative.
Yang 2020	Viral shedding and immunological features of 35 hospitalized children with Covid-19 were analyzed. 14/35 of the children had no symptoms; CT scan showed pneumonia in 32/35 children. Viral RNA was detected in fecal samples from 17/35. RNA was found in fecal samples up to 33 days after case detection.
Young 2020	SARS-CoV-2 Virus was detectable in the stool of 4 of 8 hospitalized patients.
Yuan 2020	A retrospective case note survey of 2,138 paediatric patients with suspected SARS-CoV-2 infection in Wuhan Children’s Hospital included PCR tests on both throat swabs and anal swabs were available for 212 children. Viral loads detected on both throat and anal swabs available for 24 patients showed no significant difference. The findings suggested that in some children, fecal shedding may be a sign of prolonged mildly asymptomatic infection and represent the final phase of the disease.
Zhang J 2020	A small pilot sample of 14 hospitalised cases indicated agreement for the presence of COVID-19 between oropharyngeal samples and fecal samples.
Zhang T 2020	Three children with mild symptoms who were SARS‐CoV‐2 throat swab specimen negative on discharge from hospital were stool positive 10 days post- discharge.
Zhang W 2020	A small study of hospitalised COVID-19 patients indicated that RNA of SARS-CoV-2 may be shed via multiple bodily routes, and highlights that it is found in anal swabs sometimes when oral swabs show no viral RNA.
Zhang Y, Chen C, Zhu S 2020	A 2019-nCoV strain was isolated from a stool specimen of a laboratory-confirmed Covid-19 severe pneumonia case, who experienced onset on 16 January 2020 and was sampled on 1 February 2020 in China. The full-length genome sequence indicated that the virus had high-nucleotide similarity (99.98%) to that of the first isolated novel coronavirus isolated from Wuhan. In the Vero cells, viral particles with typical morphology of a coronavirus could be observed under the electron microscope.
Zhang Z, Chen C, Song Y 2020	Samples from 258 Covid-19 patients with clinical symptoms and positive PCR were collected: 93/258 stool samples were PCR positive; PCR-positivity in stool did not correlate with GI symptoms and only suggestively correlated with disease severity. Viral load tended to be higher within respiratory swab samples. Live SARS-CoV-2 was isolated from a stool specimen (Ct value 24) of a severe Covid-19 patient (date of onset 16 January 2020)(strain HLJ002/HLJ/CHN/2020). The sequence of the full-length genome of strain HLJ002 indicated that the virus had high nucleotide similarity (99.98%) to the first SARS-CoV-2 (GenBank No. NC_045512) strain isolated from Wuhan. In the Vero cells, the virus caused obvious CPE, and the virus particles with typical morphology of coronavirus could be observed under the electron microscope. [NB it is not clear if additional cultures were attempted; we assume not.]
Sewage	
Agrawal 2020	This study monitored the time course of the SARS-CoV-2 RNA concentration in raw sewage in the Frankfurt metropolitan area of Germany. 44 sewage samples were taken from three influent sources at two wastewater treatment plants, between April and August 2020. RT-qPCR was used to assess the presence and quantity of SARS-CoV-2 RNA. The correlation of this with concurrent epidemiological surveillance data was examined. Temporal dynamics were observed between different sampling points, indicating local dynamics in Covid-19 cases within the Frankfurt metropolitan area.
Ahmed 2020	Using samples collected between February and April 2020 from sewage treatment plants in Queensland, Australia, SARS-CoV-2 was detected by RT-qPCR assay, confirmed by sequencing.
Ampuero 2020	SARS-CoV-2 RNA was detected in untreated and treated wastewater samples obtained from two treatment plants in Santiago, Chile, March to June 2020.
Arora 2020	Untreated (influent), biologically treated, and disinfected wastewater samples were collected from May to August 2020 in two North Indian states; SARS-CoV-2 RNA was detected in 16/56 samples.
Betancourt 2020	In a US university campus, wastewater from a student dormitory was tested for SARS-CoV-2 RNA. Baseline tests established no SARS-CoV-2 when the students returned to campus; subsequently the virus’ RNA was detected in wastewater samples for that dormitory. The students were isolated and tested by nasopharyngeal swab PCR to identify infected individuals. The study demonstrated surveillance using wastewater testing, leading to identifying and containing an outbreak.
Chavarria-Miro 2020	Testing of 24-hour composite raw sewage samples from two large wastewater treatment plants in Spain showed that SARS-CoV-2 was detected in sewage 41 days (15 January 2020) before the declaration of the first COVID-19 case (25 February 2020) in Spain, and in frozen samples dating back to 12 March 2019. If these results are confirmed, they suggest SARS CoV-2 has been circulating longer than first thought.
Curtis 2020	This study examined the variability of SARS-CoV-2 concentrations in wastewater grab samples collected every 2 hours for 72 hours compared with corresponding 24-hour flow-weighted composite samples. The results suggest that grab samples may be sufficient to characterize SARS-CoV-2 concentrations, but additional calculations using these data may be sensitive to grab sample variability and warrant the use of flow-weighted composite sampling.
Fongaro 2020	This study analysed human sewage in Florianopolis, Brazil from late October 2019 until the Brazil lockdown March 2020. SARS-CoV-2 was detected in two samples collected independently on 27th November 2019 (5.49 ± 0.02 log genome copies/L).
Fernandez-de-Mera 2020	This study investigated how readily SARS‐CoV‐2 RNA could be detected in environmental samples collected from an isolated small rural community in Spain at a time of a high COVID‐19 prevalence (6% of the population of 883 inhabitants). Surface samples and village wastewater samples were taken: a number of these tested PCR-positive for SARS-CoV-2 RNA but two sewage samples tested negative.
Haramoto 2020	A study of the presence of SARS-CoV-2 RNA in wastewater and river water in a prefecture of Japan and compared two laboratory methods. Whilst 1 of 5 wastewater samples tested positive, no river samples tested positive for SARS-CoV-2 RNA.
Hata 2020	A study of wastewater samples over time in Japan reported that SARS-CoV-2 RNA detection frequency increased along with the number of reported cases, and was detected even at low prevalence of <1.0 per 100,000 people. Further, the detection frequency remained high even after the increase in cases stopped.
Iglesias 2020	This study measured SARS-CoV-2 RNA from a surface water source in a low-income settlement in Buenos Aires, Argentina between June and September 2020. Measurements of SARS-CoV-2 concentrations in surface water contaminated by sewage could be used to estimate changes in Covid-19 prevalence in the local community..
Lara 2020	This study in Belgium and the Netherlands investigated the use of phylogenetic analysis in routine wastewater testing samples to evaluate the diversity of SARS- CoV-2 at the community level, and compared these results with the virus diversity in patients. It showed that this method could approximate the diversity of SARS-CoV-2 viruses circulating in a community.
La Rosa 2020 b	An environmental surveillance study based on twelve influent sewage samples, collected between February and April 2020 from wastewater treatment plants in Milan and Rome, Italy showed SARS-CoV-2 RNA fragments have been identified in sewage in Italy, and suggest a novel RT-PCR test for screening of waters.
Medema 2020	SARS-CoV-2 was detected in the sewage of five sites a week after the first COVID-19 case in the Netherlands. Even at low COVID-19 prevalence sewage surveillance could be a sensitive tool to monitor the viral circulation.
Neault 2020	In this longitudinal study, the stochastic variability inherent to wastewater-based epidemiology was corrected for using multiple fecal content protein biomarkers.These normalized SARS-CoV-2 protein data correlated well with public health SARS-CoV-2 prevalence metrics.
Ong 2020	The study ran from 24th January to 4th February 2020 and involved sampling in the physical areas around three COVID-19 patients at the Singapore dedicated SARS-CoV-2 outbreak center. The toilet bowl (seat and inner surface) and sink samples were positive, suggesting that viral shedding in stool could be a potential route of transmission. Post-cleaning samples were negative, suggesting that current decontamination measures were sufficient.
Peccia 2020	In an urban area of NE USA, this study of primary sewage sludge over time reported identifying SARS-CoV-2 RNA in all the samples. Adjusted for the time lag, the virus RNA concentrations tracked the Covid-19 epidemiological curve. SARS-CoV-2 RNA concentrations were a leading indicator of community infection ahead of compiled Covid-19 testing data and local hospital admissions.
Sharif 2020	78 wastewater samples collected from 38 districts across Pakistan, 74 wastewater samples from existing polio environmental surveillance sites, 3 from drains of Covid-19 infected areas and 1 from Covid-19 quarantine center drainage, were tested for presence of SARs-CoV-2. 21 wastewater samples (27%) from 13 districts were positive by RT-qPCR. This surveillance system has potential to aid monitoring of the pandemic, but attention is needed on virus concentration and detection assay to increase the sensitivity.
Shutler 2020	Combining in vitro data, pollution analysis and a virus survivability model, based on data from 39 countries, SARS-CoV-2 can remain stable within water for up to 25 days. Country-specific risk of infection posed by faecal contaminated water is environment-dependent, with water flow and temperature as important variables.
Trottier 2020	SARS-CoV-2 RNA was assessed in samples from the inflow point of the main waste water treatment plant of Montpellier, France, spring 2020. Samples were collected 4 days before the end of lockdown (7 May 2020) up to 70 days post-lockdown (20 July 2020). Increased amounts of SARS-CoV-2 RNA were observed from mid-June on, whereas the number of new Covid-19 cases recorded in the area started increasing a fortnight later.
Wang J 2020	The study reports the presence of SARS-Cov-2 in the hospital environment, surfaces, sewage, and the staff PPE in isolation wards in a Covid-19 hospital in China. SARS-Cov-2 RNA were positive from inlets of the sewage disinfection pool and negative from the outlet of the last sewage disinfection pool but no viable virus was detected by culture.
Wang XW 2020	No live SARS-CoV was found in any sewage samples from two hospitals receiving COVID-19 patients. SARS-CoV RNA was detected in sewage concentrates of two hospitals receiving SARS patients prior to disinfection, and occasionally after disinfection.
Wurtzer 2020	An increase of SARS-CoV-2 genome units in raw wastewaters in and around Paris, France accurately followed the increase of human COVID-19 cases observed at the regional level.
Zhao 2020	Wastewater, sludge, surface water, ground water, and soil samples of municipal and hospital wastewater systems and related environments in Wuhan during the Covid-19 middle and low risk periods were tested for SARS-CoV-2 RNA and the viral copies quantified using RT-qPCR. During the middle risk period, 1 influent sample and 3 secondary treatment effluents, 2 influent samples from wastewater system of a Covid-19 hospital were SARS-CoV-2 RNA positive. 1 sludge sample collected from a Covid-19 hospital 4 during a low risk period, was positive for SARS-CoV-2 RNA.
Toilet and or Sewage	
Del Brutto 2020	SARS-CoV-2 prevalence and incidence were assessed in a rural Guatemalan village setting using serology. One month after baseline testing, 362 of 370 initially seronegative individuals were re-tested to assess incidence of seroconversion and associated risk factors. Twenty-eight of them (7.7%) became seropositive. The overall incidence rate ratio was 7.4 per 100 person months of potential virus exposure (95%CI 4.7 to 10.2). The only covariate significantly associated with seroconversion was the use of an open latrine.
Ding Z 2020	This study randomly sampled in rooms and areas in the COVID-19 designated infectious diseases hospital Nanjing, China. 4/107 surface samples tested positive: two ward door door-handles, one bathroom toilet toilet-seat cover and one bathroom door door-handle. Three were weakly positive from a bathroom toilet seat, one bathroom washbasin tap lever and one bathroom ceiling exhaust louvre. 1/46 corridor air samples tested weakly positive.
Kang 2020	An outbreak of 9 confirmed cases of Covid-19 between 26 January 2020 and 13 February 2020 in 3 vertically aligned flats in a high-rise building in Guangzhou, China, during a period of social distancing, was investigated. There were 9 infected individuals, 193 other residents of the building, and 24 members of the building's management staff. The researchers collected environmental samples and measured the drainage airflow dispersion of a tracer gas in the block to investigate the potential for a fecal aerosol transmission route of SARS-CoV-2. No evidence was found for transmission via the elevator or elsewhere. The families lived in 3 vertically aligned flats connected by drainage pipes in the master bathrooms. Both the observed infections and the locations of positive environmental samples are consistent with the vertical spread of virus-laden aerosols via these stacks and vents. After tracer gas was released though one flat’s toilet it was found in all 5 other flats monitored, supporting the possibility of fecal aerosol spread.
Ong 2020	Between 24 January and 4 February 2020, 3 patients in airborne infection isolation rooms with anterooms and bathrooms had surface environmental samples taken at 26 sites, and air samples. Samples taken after cleaning for two patients were all negative. For one patient (samples taken pre-cleaning): 13/15 room sites including air outlet fans, 3/5 toilet sites (toilet bowl, sink, and door handle) were PCR positive. Anteroom and corridor samples were negative. This patient had upper respiratory tract involvement with no pneumonia and had 2 positive stool samples for SARS-CoV-2 on RT-PCR despite not having diarrhea.
Waterways	
Guerrero-Latorre 2020	This study assessed the presence of SARS-COV-2 in urban streams from a low sanitation context i.e. highly impacted by sewage. Three river locations along the urban rivers of Quito, Ecuador were sampled on the 5 June 2020 during a peak of Covid-19 cases. SARS-CoV-2 RNA was detected in all samples, at levels similar to those in wastewater from cities during outbreaks.

**Table 5.** Viral culture using fecal samples (n=6 studies).

Study (n=6)	Method	Viral culture of fecal samples n successful/n attempted	Notes	Methodological issues
Jeong 2020	Specimens were used to inoculate Vero cells; these were cultured in Eagle's minimal essential medium with 8% heat-inactivated fetal bovine serum and antibiotics. Cells were monitored daily for 4 days to examine the cytopathic effects. To confirm virus isolation, RT-PCR was performed on supernatants from infected cell cultures.	0/3	The same study was able to isolate viable SARS-CoV-2 from naso/ oropharyngeal swabs and saliva of COVID-19 patients, as well as nasal washes of ferrets inoculated with patient urine or stool.	CPE, not plaque assays were cited as evidence of virus replication. The low RNA levels, late disease stage, and high antibody render it unlikely this study resulted in cultured virus.
Kim JM 2020	For the cell inoculation, cells were cultured from the CaCo-2 cell line (derived from human epithelial colorectal adenocarcinoma cells) in Dulbecco modified Eagle medium supplemented with 20% fetal bovine serum and 1% penicillin, and were incubated at 37°C, 5% CO2. The cells were cultured for 5 days and then harvested. To evaluate the viral replication process, RNA from the secondary inoculation cell culture supernatant was extracted and assessed for the presence of SARS-CoV-2 using real-time RT-PCR.	0/13	Collection of samples was reported as date of hospital admission (not reported in relation to date of symptom onset).	Enough genomes by PCR to grow virus if it was there. Concerned they couldn’t detect any virus replication. Some should have been positive, but an unusual, indirect assay was used.
Wang W, Xu Y 2020	No details	2/4	Electron microscopy was performed to detect live virus.	Electron microscopy can identify virions but does not demonstrate viral viability.
Wölfel 2020	Vero E6 cells were seeded on a 24-well plate at 3.5 × 10^5 cells/mL in Dulbecco modified Eagle medium containing 1% sodium pyruvate, 1% nonessential amino acids, 1% l-glutamine, and 10% fetal calf serum one day prior to inoculation. Cells were observed daily for cytopathogenic effects for 6 days. Every 2 days or upon observation of cytopathogenic effects, 50 μl of cell culture supernatant was subjected to viral RNA extraction and SARS-CoV-2 specific real-time RT-PCR using the SARS-2-CoV E assay.	0/13	Culture of the virus was attempted on multiple occasions from 13 fecal samples from 4 patients with high viral loads between day 6 and day 12 of infection.	Had positive controls showing an RNA increase in culture, absence in fecal samples suggests no infectious virus. Robust study, clearly illustrating the problem detecting infectious material after seroconversion.
Xiao F, Sun J 2020	The cytopathic effect in Vero E cells was observed 2 days after a second-round passage. Negatively stained culture supernatant was visualized by transmission electron microscopy. Viral particles that were visible were spherical and had distinct surface spike protein projections, consistent with a previously published SARS-CoV2 image. Viral nucleic acid was collected from cell culture supernatant, whole genome sequencing identified SARS- CoV-2.	2 cases	Virus culture was attempted from an unreported number of specimens and cases, successful in two cases.	The CPE shown in the figure is not very convincing; it does not show plaques. Not immunostained, no evidence by PCR of growth.
Zhang Y 2020	Vero cells were used for viral isolation from stool samples (no other methods described). Full-length genome sequence on the one specimen was identified using ABI 3130 Genetic Analyzer. Genome sequence indicated high nucleotide similarity (99.98%) to first isolated novel coronavirus from Wuhan.	1	Isolated the virus from the stools of one severe hospitalised COVID-19 pneumonia case. The number of samples taken is unclear.	No evidence of infectious material; EM is not proof of this.

## Discussion

The evidence from 110 relevant studies supports a potential role of orofecal transmission of SARS-CoV-2. Fecal shedding of SARS-CoV-2 RNA has been reported in 96% of the included observational studies, often for relatively long durations. Three studies reported the culture of SARS-CoV-2 using fecal samples, but requisite methods to confirm viral growth were lacking. One study demonstrated viral isolation from rectal tissue of a COVID-19 patient. Studies in hospitals show the presence of SARS-CoV-2 RNA at and around toilets and toilet rooms; there is evidence that disinfectant cleaning leaves no SARS-CoV-2 RNA detectable. Many studies report identifying SARS-CoV-2 RNA in sewage and wastewaters, but viral culture from such sources has not been demonstrated, and therefore the detection in sewage and wastewaters can be useful as a surveillance tool.

Experimental models of the human intestinal epithelium show that SARS-CoV-2 can infect this tissue and replicate, supporting the rationale for the human GI tract as a possible transmission route ^4– 6^). Zang 2020 demonstrated that human enterocytes express high ACE2 receptor levels, supporting viral invasion at these sites ^4^. Zang 2020 and Lamers 2020 *et al.* showed that SARS-CoV-2 productively infected human small intestinal organoids ^4, 5^. Zhou J 2020 *et al.* and co-workers showed active replication of SARS-CoV-2 in human intestinal organoids, and isolated infectious virus from the stool specimen of a patient with diarrheal COVID-19 ^6^. Yao *et al.* investigated the mutation spectrum, replication dynamics, and infectivity of 11 patient-derived SARS-CoV-2 isolates in diverse cell lines; the authors report that "three of our viral isolates were extracted from stool samples (two of which were very potent) indicating that viable SARS-CoV-2 particles could be found in stool samples" ^7^.

MERS-CoV has been shown to infect human primary intestinal epithelial cells, small intestine explants and intestinal organoids ^8^. MERS-CoV has been detected in 42% of milk samples collected from lactating camels where it can survive for a prolonged period. A study of human primary intestinal epithelial cells and small intestine explants of MERS-CoV patterns identified the viral replication intermediates in stool specimens. MERS-CoV was found to be resistant to fed-state gastrointestinal fluids but less tolerant to the high acidic fasted-state gastric fluid.

Prolonged excretion of coronaviruses in feces was first observed in 1977 ^9^. In the SARS-CoV-1 outbreak in 2002-03, a significant portion of patients had enteric involvement. In the Toronto outbreak in 2003, 6% of 144 patients had diarrhoea on presentation ^10^. Among 138 patients with SARS in Hong Kong, 20% presented with watery diarrhoea and 38% had symptoms of diarrhoea during the illness. Intestinal biopsy specimens showed the presence of active viral replication, and SARS-CoV RNA was detected in the stool of some patients for more than ten weeks after symptom onset ^11^. A retrospective study on specimens from 154 patients in Hong Kong with laboratory-confirmed SARS found the viral load to be the highest in stool specimens ^12^. Up to 70% of 75 patients in a community outbreak in Hong Kong developed watery diarrhoea ^13^. This outbreak was linked to a faulty sewage system in the Amoy Gardens apartment complex, further suggesting orofecal transmission might be a route for transmission ^14^.

The human gastrointestinal tract can act as a primary infection site for SARS-CoV. Ding *et al.* used a monoclonal antibody specific for the SARS‐CoV nucleoprotein, and probes for the RNA polymerase gene fragment in four patients who died from SARS‐CoV-1 ^15^. Virus was detected in the stomach, small intestine, distal convoluted renal tubule, sweat gland, parathyroid, pituitary, pancreas, adrenal, liver and cerebrum. The authors discussed that viruses in contaminated food and water may enter the human body through epithelial cells covering the surface of the gastrointestinal tract, although there was no direct evidence to show that food‐borne transmission had occurred. A study from the sewage of two hospitals receiving SARS patients in Beijing found no infectious SARS-CoV contamination in any of the samples collected but did detect the nucleic acid in the sewage from the two hospitals before disinfection - providing further evidence that SARS-CoV-1 can be excreted by feces into the sewage system ^16^.

Transmission of coronaviruses via the feces is established among animals: feline coronavirus, for instance, is typically shed in feces of healthy cats and transmitted by the orofecal route to other cats ^17^. Pigs are also infected by the transmissible gastroenteritis coronavirus via the orofecal ^18^. Bat coronavirus infects the gastrointestinal and respiratory tracts of bats, seemingly without causing disease ^19^. Transmission following exposure to camel feces has also been considered biologically plausible ^20^.

There is evidence that SARS-CoV-2 can survive adverse conditions in the gastrointestinal system. It has been identified in endoscopic specimens of the oesophagus, stomach, duodenum, and rectum of COVID-19 patients; substantial amounts of SARS-CoV-2 RNA have been consistently detected in stool specimens, and evidence suggests that SARS-CoV-2 can survive the adverse conditions in the gastrointestinal system. Heavy glycosylation of the large spike S protein has been shown to lead to resistance to the proteases, the low pH and bile salts found in the gastrointestinal system. Some gastric processes may actually facilitate viral entry into the enterocytes: in bovine coronavirus, one specific site on the S glycoprotein has to be cleaved by an intracellular protease or trypsin to activate viral infectivity and cell fusion ^21^.

Evidence of ingestion, penetration of enterocytes and excretion of live SARS-CoV-2 is possible; however, this working hypothesis requires testing by conducting case-control studies during the investigation of outbreaks, following a set protocol. For such investigations, cases of COVID-19 (categorised by symptom presence and severity) either fecally excreting virions or not (cases and contacts) and controls would be healthy matches. Exposure to potentially fecally contaminated materials and protective measures taken would be elicited at interview. To minimise the play of recall and ascertainment bias, interviewers should be blind to fecal excretion status and the interview should take place as soon as possible after the event. Viability of fecal isolates and their possible pathogenicity should be tested in outbreaks.

***Strengths and limitations.*** This review is limited by the quality of included studies: many were small and did not provide a protocol that established a priori methods. Studies were often poorly reported and often did not take biases into account. Reporting is often heterogeneous and essential information such as symptom onset and cycle threshold values were often missing. We do not have information on publication bias, but the current urgency to understand SARS-CoV-2 may have an impact on research, with unknown implications and a tendency to publish those studies with positive results. It is likely we missed some studies, but we plan to keep updating this review. Furthermore, our judgments of quality are to some extended subjective and open to disagreement. This does not, however, undermine our overall assessment of the quality of the included studies. We perceive that standardization of methods in this area would improve the quality of the research. Some of these limitations increase uncertainties and prevent firm conclusions being drawn; however, this body of research provides largely consistent evidence on the main conclusions that SARS-CoV-2 is excreted fecally, is found in sewage and can be cultured from fecal samples.

## Conclusion

Observational and mechanistic evidence as well as established animal orofecal transmission of coronaviruses suggests SARS-CoV-2 can infect and be shed from the human gastrointestinal tract. Future studies should test this hypothesis rigorously to allow the development of appropriate public health measures.

## Data availability

### Underlying data

All data underlying the results are available as part of the article and no additional source data are required.

### Extended data

Figshare: Extended data for SARS-CoV-2 and the role of orofecal transmission: a systematic review, https://doi.org/10.6084/m9.figshare.14247470.v1 ^2^.

This project contains the following extended data:

- Appendix 1: Updated protocol
- Appendix 2: Search strategy
- Appendix 3: References to included studies.

### Reporting guidelines

Figshare: PRISMA checklist for ‘SARS-CoV-2 and the role of orofecal transmission: a systematic review’, https://doi.org/10.6084/m9.figshare.14247470.v1 ^2^.

Data are available under the terms of the Creative Commons Attribution 4.0 International license (CC-BY 4.0).
