# Supplementary material for: SARS-CoV-2 and the role of orofecal transmission: a systematic review
Source: F1000Res. 2021 Nov 2;10:231. Originally published 2021 Mar 24. [Version 2] doi: 10.12688/f1000research.51592.2 (PMC8749895; doi:10.12688/f1000research.51592.2)
Supplement: Peer review history

# Peer review: Reviewer response for version 2

**DOI:** 10.5256/f1000research.78110.r98695

**Published:** 2022-01-07

**Authors:** Mark D. Sobsey

**Affiliations:** 1 Department of Environmental Sciences and Engineering, Gillings School of Global Public Health, Chapel Hill, NC, USA; 2 University of North Carolina at Chapel Hill, Chapel Hill, USA

I have read the revised article and the response of the manuscript authors. I agree with the authors' response and the changes in the manuscript. The authors now make clear that so far, there is no evidence for the presence of infectious SARS-CoV-2 in sewage, other wastewater and environmental waters. I support this position. In my judgement, I now consider the article acceptable without reservations. 

 Furthermore, the authors and other readers of the manuscript may be interested to know that I recently published an invited review article in the Journal of Water and Health, a journal of the international Water Association, that is entitled: "Absence of virological and epidemiological evidence that SARS-CoV-2 poses COVID-19 risks from environmental fecal waste, wastewater and water exposures' (Sobsey, 2021 ^1^).

 My JWH article further supports the assessments and conclusions of the authors of this F1000 Research article.

Are the rationale for, and objectives of, the Systematic Review clearly stated?

Yes

Is the statistical analysis and its interpretation appropriate?

Partly

Are sufficient details of the methods and analysis provided to allow replication by others?

Partly

Are the conclusions drawn adequately supported by the results presented in the review?

Partly

Reviewer Expertise:

NA

I confirm that I have read this submission and believe that I have an appropriate level of expertise to confirm that it is of an acceptable scientific standard.

## References

[1] : Absence of virological and epidemiological evidence that SARS-CoV-2 poses COVID-19 risks from environmental fecal waste, wastewater and water exposures. *Journal of Water and Health* .2021; 10.2166/wh.2021.182 10.2166/wh.2021.182 35100160

# Peer review: Reviewer response for version 2

**DOI:** 10.5256/f1000research.78110.r98696

**Published:** 2021-11-12

**Authors:** Jennifer Audsley

**Affiliations:** 1 Department of Infectious Diseases, The University of Melbourne at the Peter Doherty Institute for Infection and Immunity, Melbourne, Australia

Thanks to the authors for the responses to my queries. The issues have been addressed satisfactorily.

Are the rationale for, and objectives of, the Systematic Review clearly stated?

Yes

Is the statistical analysis and its interpretation appropriate?

Partly

Are sufficient details of the methods and analysis provided to allow replication by others?

Yes

Are the conclusions drawn adequately supported by the results presented in the review?

Partly

Reviewer Expertise:

I am a Clinical Research Fellow with a major research interest in HIV-Hepatitis co-infection, and since 2020 also COVID-19 infection

I confirm that I have read this submission and believe that I have an appropriate level of expertise to confirm that it is of an acceptable scientific standard.

# Peer review: Reviewer response for version 1

**DOI:** 10.5256/f1000research.54778.r89625

**Published:** 2021-08-12

**Authors:** Jennifer Audsley

**Affiliations:** 1 Department of Infectious Diseases, The University of Melbourne at the Peter Doherty Institute for Infection and Immunity, Melbourne, Australia

Heneghan *et al.* present a systematic review of SARS-CoV-2 and the role of orofecal transmission. They included 110 relevant studies (including 35 reviews) from searches up until 20 ^th^ December 2020. Potential orofecal transmission of SARS-CoV-2 is an important public health issue. They found the observational evidence suggested SARS-CoV-2 infects and is shed from the gastrointestinal tract (GIT) and with further testing could allow the development of appropriate public health measures. This systematic review has followed the PRISMA guidelines including developing a review protocol and completing the PRISMA checklist. All underlying data is available and extended data has been uploaded onto Figshare. This review is very well-written and the references relevant. The authors have undertaken this review thoroughly, with the largely low-moderate data available. There are, however, some points that should be addressed to strengthen the review and conclusions.

- The aim was to systematically review evidence on orofecal SARS-CoV-2 transmission while the conclusion focuses on SARS-CoV-2 fecal excretion and presence in sewage, which is not evidence of transmission. Detection of SARS-CoV-2 in stool samples and in ecological settings is important but it should be noted that was not the aim of this review. Viral culture data from six studies is included but this is a small minority of studies, and as the authors note due to methodological issues should be interpreted with caution.

- Examining the ecological studies and review is very worthwhile but none address the issues of orofecal SARS-CoV-2 transmission. Perhaps ecological factors are a secondary outcome?

- While the authors do note in the results that some reviews included overlapping studies, there is no figure/percentage given for this. How much overlap in total is there, is the overlap greater for cohort/case series or ecological studies? What is the quality of the overlapping studies – are they over-representing the low-quality data?

- What is the breakdown for included reviews between cohort/case series and ecological studies? This is provided for primary studies in Figure 1 but I can’t find it for the reviews.

- Have you considered a sub analysis of the studies in children?

- Is gender reported routinely across the studies?

- How many/percentage of the studies are only on MedRxiv? Consider including breakdown by primary/review, cohort-case series/ecological. This could be a source of bias.

Minor:

- Table 1 pages 10, 11 & 12 – total patient number from the cohort/case series (9081) appears in the “sewage” header rows.

Are the rationale for, and objectives of, the Systematic Review clearly stated?

Yes

Is the statistical analysis and its interpretation appropriate?

Partly

Are sufficient details of the methods and analysis provided to allow replication by others?

Yes

Are the conclusions drawn adequately supported by the results presented in the review?

Partly

Reviewer Expertise:

I am a Clinical Research Fellow with a major research interest in HIV-Hepatitis co-infection, and since 2020 also COVID-19 infection

I confirm that I have read this submission and believe that I have an appropriate level of expertise to confirm that it is of an acceptable scientific standard, however I have significant reservations, as outlined above.

# Response

**Published:** 2021-09-30

**Authors:** Elizabeth Spencer

**Affiliations:** University of Oxford , UK

Thank you for these helpful suggestions to improve the review. Please see our changes made, noted below. 

 1. Added: typically quantified by the number of virions, gene copies or equivalents, or by antigen indicators of viral load.

 2. Added to the Discussion: these studies do not provide an estimate of infectious virus concentration; nor has the infectious dose for humans been established, so at this point it is unwarranted to deduce a human transmission risk based on this small number of virus-positive fecal samples.

 3. Changed Discussion sentence: Many studies report identifying SARS-CoV-2 RNA in sewage and wastewaters, but viral culture from such sources has not been demonstrated, so there is no evidence of infection risk from those sources; however, detection of SARS-CoV-2 RNA in sewage and wastewaters can be useful as a surveillance tool.

 4. Added: However, quantitative data on infectiousness and the consequent likelihood of transmission from orofecal contamination is not available. Whilst SARS-CoV-2 RNA is observed in sewage and wastewaters, there is no evidence of infectiousness in these sources and a transmission risk from these sources is therefore unlikely. To properly assess these risks, quantitative data on infectious virus are needed, along with information on likely infectious dose in humans.

 5. Added to Discussion: We note the possibility that some cases may be reported in more than one publication, but there is no adequate means by which to identify this.

 6. Unclear reporting in the reviews did not allow full analysis of their included studies. To do this we could revisit the full text of their included studies, but this is beyond the scope of this review. Inclusion of these reviews represents a level of interest in the field but necessarily they will overlap in scope and therefore we do not analyse their findings in detail.

 7. Thank you for the suggestion to further sub analyse the data; at this stage there are insufficient data for such sub analyses including among children.

 8. Unfortunately there are several studies in which sex of participants is not reported and so we were limited by this. 

 9. This was a very interesting suggestion to sub-analyse according to preprint publication and examine possible publication biases; this is unfortunately beyond the scope of this review, not least because this changes over time. The next time we update this review we hope to have a better breakdown of those fully published, and hopefully a better bank of evidence to analyse.

 10. Thank you for noting the anomaly in the table, we have checked and rectified and hopefully this will all be in the right place now. 

 Thank you for these comments.

# Peer review: Reviewer response for version 1

**DOI:** 10.5256/f1000research.54778.r82122

**Published:** 2021-04-07

**Authors:** Mark D. Sobsey

**Affiliations:** 1 Department of Environmental Sciences and Engineering, Gillings School of Global Public Health, Chapel Hill, NC, USA; 2 University of North Carolina at Chapel Hill, Chapel Hill, USA

This article is of generally high quality, but improvements are suggested in some areas, including in the conclusions. Starting from the beginning of the article here are some points that should be clarified or improved:

1. In Box 1. Terminology, Viral load is defined in a rather vague and insufficiently quantitative way. Viral load can be quantified on the basis of virions (physical particles (as seen by electron microscopy), gene copies or equivalents based on RNA units or CT values, antigen units such viral proteins quantified in different ways, including immunofluorescence intensity observed in infected tissues and as infectious units for replication competent virus based on titers in susceptible cell cultures or experimental animals. There is value in focusing on quantified "viral load" in human gastrointestinal tissues, intestinal contents and fecally contaminated materials such as fomites, wastewater (e.g., sewage) and water (such as recreational, agricultural irrigation and drinking waters). These nuances in "viral load" are important in interpreting and communicating the transmission-relevant results reported in the research studies reviewed..
2. The data found in the study and provided in Table 1 indicate that only a few studies (5 according to table 1) attempted to detect infectious or replication-competent SARS-CoV-2 in either feces, sewage or a related sample, of which only 3 reported the presence of infectious virus. Further, infectious virus results are reported as "yes" or "no" (presence or absence) per sample. The data for infectious virus are not quantified in typical units such as plaque-forming units or TCID-50 units. Therefore, the concentrations of the detected viruses are unknown. Such lack of quantitative data on infectious virus concentration makes it impossible to determine if sufficient infectious virus is present to pose a transmission risk from human exposure. This point should be mentioned in the overall presentation of the infectious virus results. Furthermore, we do not know the infectivity dose-response relationship of SARS-CoV-2 for human hosts, such an estimate of 50% infectious dose. Unless we know what virus concentration is in samples such as feces or sewage/wastewater and unless we know what the likelihood of human infection is from exposure to known concentrations of infectious virus, it is probably unjustified to suggest that the presence of an unknown concentration of infectious virus in a very small number of reported virus-positive fecal samples poses a human health risk from exposure. While infectious virus presence in a few samples is noteworthy, the lack of quantitative infectivity data makes it impossible to judge that there is plausible human health exposure risk from this infectious virus-positive fecal matter of unknown concentration. The best one can say is that the risk of infection or other health effects from exposure is not zero, but is also not quantifiable due to lack of data on virus concentration in feces and lack of knowledge of human infectivity dose-response for infection or other health endpoints. 
3. For sewage and water, the lack of data on virus infectivity in such samples makes it impossible to determine if these samples pose risks of human infection from exposure. Although some data on concentrations of viral RNA are reported based on estimated gene copies or CT values, the quantitative relationship of virus infectious units to RNA concentration units is highly variable and therefore uncertain This is especially so for samples that have been in the environment for unknown and variable periods of time since the feces were shed from a human host. This is due to the variable and difficult-to-quantify effects of exposure time, temperature, microbial activity, predation and other environmental stressors on virus survival. These factors are not mentioned in the paper, even though we know they are important for determining the quality and validity of the results for infectious virus presence and concentrations. The last sentence of the first paragraph of the Discussion section does not adequately address the issue of lack of evidence of virus culture in wastewater and sewage and its implications for human health risks from sewage. Instead it sidesteps to the potential value using SARS-CoV-2 RNA in sewage and wastewater as a surveillance tool. This seems to miss the main point of assessing the evidence for human health risk from exposure to sewage or wastewater.In the "strengths and limitations section of the Discussion section, the authors state "however, this body of research provides largely consistent evidence on the main conclusions that SARS-CoV-2 is excreted fecally, is found in sewage and can be cultured from fecal samples." While there there is some evidence that unknown but probably low concentrations of infectious SARS-CoV-2 have been occasionally found in excreted feces, they have not been found in sewage or wastewater. The wording of the above sentence conflates feces with wastewater and sewage, even though there is only limited and poor evidence for infectious virus in the latter and no evidence for it in sewage or wastewater. I recommend that the sentence be revised to avoid conflating these rather separate exposure sources of feces versus sewage and wastewater.
4. The Conclusion sentence is short and limited in scope. It does not adequately address the issue of whether sufficient infectious SARS-CoV-2 is or is not likely to be present in feces or wastewater and sewage to pose human health risks from exposure. The available evidence seems to indicate that such risks are not possible to assess or quantify without better and more rigorous and as well as more quantitative data that is still not available.

Are the rationale for, and objectives of, the Systematic Review clearly stated?

Yes

Is the statistical analysis and its interpretation appropriate?

Partly

Are sufficient details of the methods and analysis provided to allow replication by others?

Partly

Are the conclusions drawn adequately supported by the results presented in the review?

Partly

Reviewer Expertise:

I am an environmental and public health virologist and microbiologist with expertise in infection protection and control and water-sanitation and hygiene, as well as in infectious disease epidemiology in the context of environmental exposure risks and disease transmission of human pathogens .

I confirm that I have read this submission and believe that I have an appropriate level of expertise to confirm that it is of an acceptable scientific standard, however I have significant reservations, as outlined above.

# Response

**Published:** 2021-09-30

**Authors:** Elizabeth Spencer

**Affiliations:** University of Oxford , UK

Thank you for these helpful suggestions to improve the review. Please see our changes made, noted below. 

 1. Added: typically quantified by the number of virions, gene copies or equivalents, or by antigen indicators of viral load.

 2. Added to the Discussion: these studies do not provide an estimate of infectious virus concentration; nor has the infectious dose for humans been established, so at this point it is unwarranted to deduce a human transmission risk based on this small number of virus-positive fecal samples.

 3. Changed Discussion sentence: Many studies report identifying SARS-CoV-2 RNA in sewage and wastewaters, but viral culture from such sources has not been demonstrated, so there is no evidence of infection risk from those sources; however, detection of SARS-CoV-2 RNA in sewage and wastewaters can be useful as a surveillance tool.

 4. Added: However, quantitative data on infectiousness and the consequent likelihood of transmission from orofecal contamination is not available. Whilst SARS-CoV-2 RNA is observed in sewage and wastewaters, there is no evidence of infectiousness in these sources and a transmission risk from these sources is therefore unlikely. To properly assess these risks, quantitative data on infectious virus are needed, along with information on likely infectious dose in humans.
